# Enhancing immunotherapy efficacy in colorectal cancer: targeting the FGR-AKT-SP1-DKK1 axis with DCC-2036 (Rebastinib)

**DOI:** 10.1038/s41419-024-07263-8

**Published:** 2025-01-09

**Authors:** Xiguang Chen, Qiting Zeng, Liyang Yin, Bingru Yan, Chen Wu, Jianbo Feng, Ying Wu, Jun He, Wenjun Ding, Jing Zhong, Yingying Shen, Xuyu Zu

**Affiliations:** 1 The First Affiliated Hospital, Cancer Research Institute, Hengyang Medical School, University of South China, Hengyang, Hunan 421001 PR China; 2 Hunan Province Key Laboratory of Tumor Cellular & Molecular Pathology, Cancer Research Institute, Hengyang Medical School, University of South China, Hengyang, Hunan 421001 China; 3 The First Affiliated Hospital, Gastrointestinal Surgery Department, Hengyang, Hunan 421001 PR China; 4 The First Affiliated Hospital, Department of Clinical Laboratory Medicine, Hengyang, Hunan 421001 PR China; 5 Central Hospital of Hengyang City, Oncology Department, Hengyang, Hunan 421001 PR China; 6 The First Affiliated Hospital, Department of Ultrasound Imaging, Hengyang Medical School, University of South China, Hengyang, 421001 China; 7 The Nanhua Affiliated Hospital, Department of Spine Surgery, Hengyang Medical School, University of South China, Hengyang, Hunan 421001 China; 8 Hunan Provincial Clinical Medical Research Center for Drug Evaluation of Major Chronic Diseases, University of South China, Hengyang, Hunan 421001 China

**Keywords:** Colorectal cancer, Immunosurveillance

## Abstract

This research demonstrates that DCC-2036 (Rebastinib), a potent third-generation tyrosine kinase inhibitor (TKI), effectively suppresses tumor growth in colorectal cancer (CRC) models with functional immune systems. The findings underscore the capacity of DCC-2036 to enhance both the activation and cytotoxic functionality of CD8^+^ T cells, which are crucial for facilitating anti-tumor immune responses. Through comprehensive multi-omics investigations, significant shifts in both gene and protein expression profiles were detected, notably a marked decrease in DKK1 levels. This reduction in DKK1 was linked to diminished CD8^+^ T cell effectiveness, correlating with decreased FGR expression. Moreover, our findings identify FGR as a pivotal modulator that influences DKK1 expression via the PI3K-AKT-SP1 signaling cascade. Correlative analysis of clinical specimens supports the experimental data, showing that increased levels of FGR and DKK1 in CRC tissues are associated with inferior clinical outcomes and reduced efficacy of immunotherapeutic interventions. Consequently, targeting the FGR-AKT-SP1-DKK1 pathway with DCC-2036 could potentiate immunotherapy by enhancing CD8^+^ T cell functionality and their tumor infiltration. This strategy may contribute significantly to the refinement of therapeutic approaches for CRC, potentially improving patient prognoses.

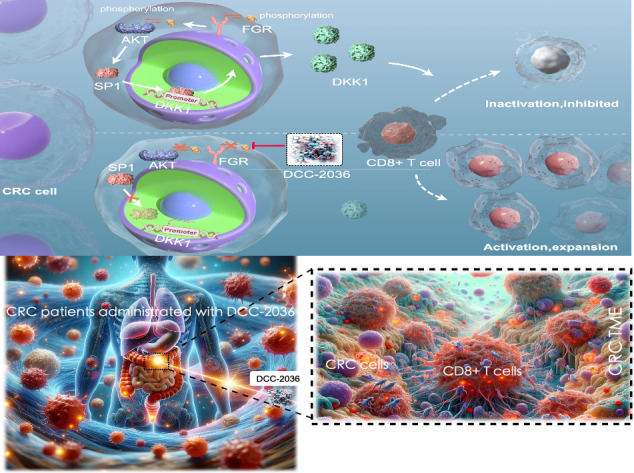

## Background

CRC is the third most prevalent malignancy globally and stands as the second primary contributor to cancer-associated mortality, responsible for approximately 9.4% of all cancer deaths, trailing only lung cancer, which accounts for 18% [1, 2]. Despite advancements in cancer research, the five-year survival rate for individuals with advanced stages of CRC remains below 13% [3]. This underscores the critical need for continued research into CRC, considering its significant prevalence and mortality rates. An in-depth understanding of the epidemiology, risk factors, and molecular mechanisms of CRC is crucial. Advances in these areas could significantly improve prevention strategies, early detection, and treatment options, thereby enhancing patient outcomes and reducing the global impact of CRC.

The emergence of immune checkpoint blockade (ICB) therapies has revolutionized the treatment of CRC. The FDA has approved ICB treatments for unresectable or metastatic CRC that display high microsatellite instability or mismatch repair deficiencies, irrespective of prior chemotherapy [4, 5]. Enhanced CD8^+^ T-cell presence in CRC correlates with improved responses to ICB [6, 7]. However, the majority of CRCs are classified as ‘cold tumors’ because of their low levels of T-cell infiltration, which generally results in suboptimal responses to immunotherapy. To tackle this challenge, it is essential to cultivate a strong immunological landscape abundant in CD8^+^ T-cells, which will enhance the tumor’s responsiveness to ICB [8]. An in-depth investigation into the biological processes that facilitate tumor infiltration and enhance CD8^+^ T-cell activity is crucial for advancing the efficacy of CRC treatments and immunotherapeutic strategies [9].

DCC-2036, a novel third-generation TKI, selectively engages ABL1 and shows significant efficacy in murine models of Bcr-Abl T315I chronic myeloid leukemia as well as in primary cells derived from patients harboring the T315I mutation [10, 11]. It also shows potential against imatinib-resistant hypereosinophilic syndrome cells with the T674I FIP1L1-PDGFR mutation and AXL-overexpressing triple-negative breast cancers [12–14]. Furthermore, DCC-2036 reduces macrophage infiltration [14, 15] and exhibits anti-angiogenic effects in breast cancer models [16], while it enhances survival in glioma models by inhibiting Tie-2 mediated pathways [15]. The broad efficacy of DCC-2036 across various cancer models highlights its potential as a targeted therapy, justifying further exploration of its underlying mechanisms and potential therapeutic applications.

Our research primarily assessed DCC-2036’s capacity to inhibit tyrosine kinases (TKs) that are frequently overexpressed or activated in CRC, such as SRC, RAF, VEGFR2, MET, and FGR [11, 17–20]. We hypothesized that DCC-2036 would be an effective treatment for CRC. The findings demonstrated that DCC-2036 exhibited greater efficacy in immunocompetent mice compared to those with immunodeficiencies, implying a mechanism reliant on T-cell-mediated immunity, as evidenced by heightened CD8^+^ T-cell infiltration and activity within tumors in vivo.

A significant discovery from our research was the identification of the non-receptor tyrosine kinase FGR as an essential target of DCC-2036 in CRC. Further studies indicated that the impact of DCC-2036 on CRC extends beyond direct tumor cell inhibition, as it profoundly influences the immune microenvironment. Specifically, by targeting FGR, DCC-2036 modulates the PI3K-AKT-SP1 signaling cascade, which subsequently affects DKK1 expression levels, ultimately enhancing the activation and cytotoxic functionality of CD8^+^ T cells. This modulation underscores the pivotal role of DCC-2036 in reshaping the immune landscape within the tumor, suggesting its potential to boost the efficacy of immunotherapeutic strategies in CRC.

## Results

### T-cell-mediated immunity enhances the efficacy of DCC-2036 in CRC

**Fig. 1:**
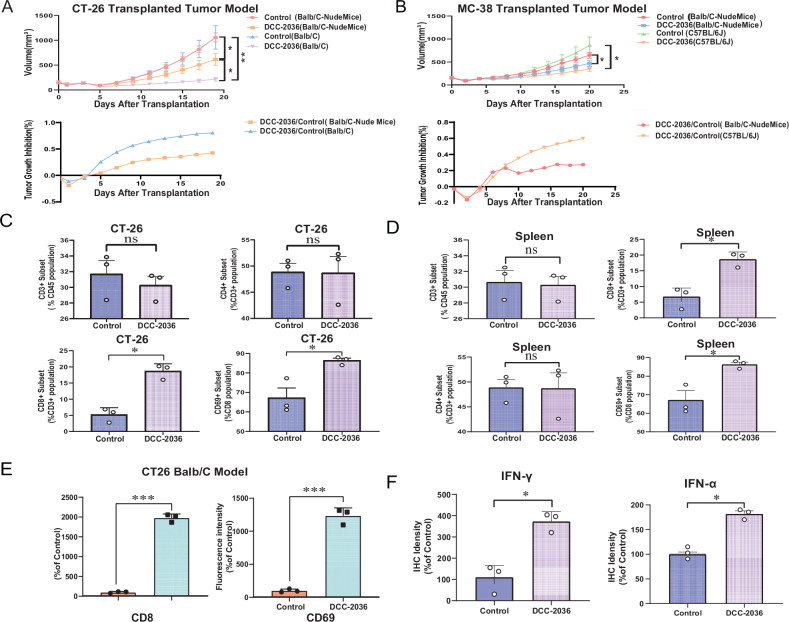
Analyzing the impact of DCC-2036 on CRC transplanted tumor: tumor growth inhibition, lymphocyte variation in CRC transplanted tumor. **A** Growth analysis in CT-26 transplanted tumor models. Quantitative growth and inhibitory curves in Balb/C and Balb/C Nude mice treated with DCC-2036 (50 mg/kg) or vehicle control. Treatments were administered via oral gavage every other day. **B** Growth analysis in MC-38 transplanted tumor models. Quantitative growth and inhibitory curves in C57BL/6 J and Balb/C Nude mice treated with DCC-2036 (50 mg/kg) or vehicle control. Treatments were administered via oral gavage every other day. **C** Bar chart showing the distribution of CD8^+^ (%CD3^+^), CD4^+^(% CD3^+^), and CD69^+^(% CD8^+^) T cells in CT-26 xenografts across different treatment groups. Data expressed as mean ± SD, analyzed using Student’s t-test (*P < 0.05, ns: not significant). **D** Statistical Analysis of T Cell Subtypes: Bar chart depicting the percentage of various T cell subtypes within tumor-bearing mice spleens. Data expressed as mean ± SD, analyzed using Student’s t-test (*P < 0.05, ns: not significant). **E** Immunofluorescence Microscopy for CD8 and CD69, bar graphs quantifying fluorescence intensity, indicating expression levels of CD8 and CD69, analyzed using Student’s t-test (***P < 0.001). **F** Immunohistochemistry Staining of IFN-α and IFN-γ: bar graphs quantifying fluorescence intensity, indicating expression levels of IFN-α and IFN-γ, analyzed using Student’s t-test (***P < 0.001).

Our study shows that DCC-2036 effectively stops the growth of CT-26/MC-38 tumors in subcutaneous tumor models. We used immunocompetent (Balb/C, C57BL/6 J) and immunocompromised (Balb/C Nude) mice for this experiment. Notably, in immunocompetent mice, the tumors treated with DCC-2036 showed extensive areas of necrosis, indicating a potent anti-tumor response, which underscores the role of T-cell-mediated immunity in enhancing the drug’s efficacy (Fig. 1A-B, Fig. S1A-B).

Flow cytometry analyses revealed a marked increase in CD8^+^ T cells and enhanced cytolytic activity (CD69 expression) within tumors and spleens of immunocompetent mice, while changes in CD4^+^ cells were minimal (Fig. 1C-D, Fig. S1C-E). Supporting these findings, immunohistochemistry and immunofluorescence studies displayed heightened immunoreactivity for CD8, CD69, IFN-α, and IFN-γ in the DCC-2036 treated group, relative to controls (Fig. S2A-B).

### Highlighting DKK1’s regulatory influence on CD8^+^ T cell dynamics through comprehensive multi-omics analysis of DCC-2036’s impact on CRC

**Fig. 2:**
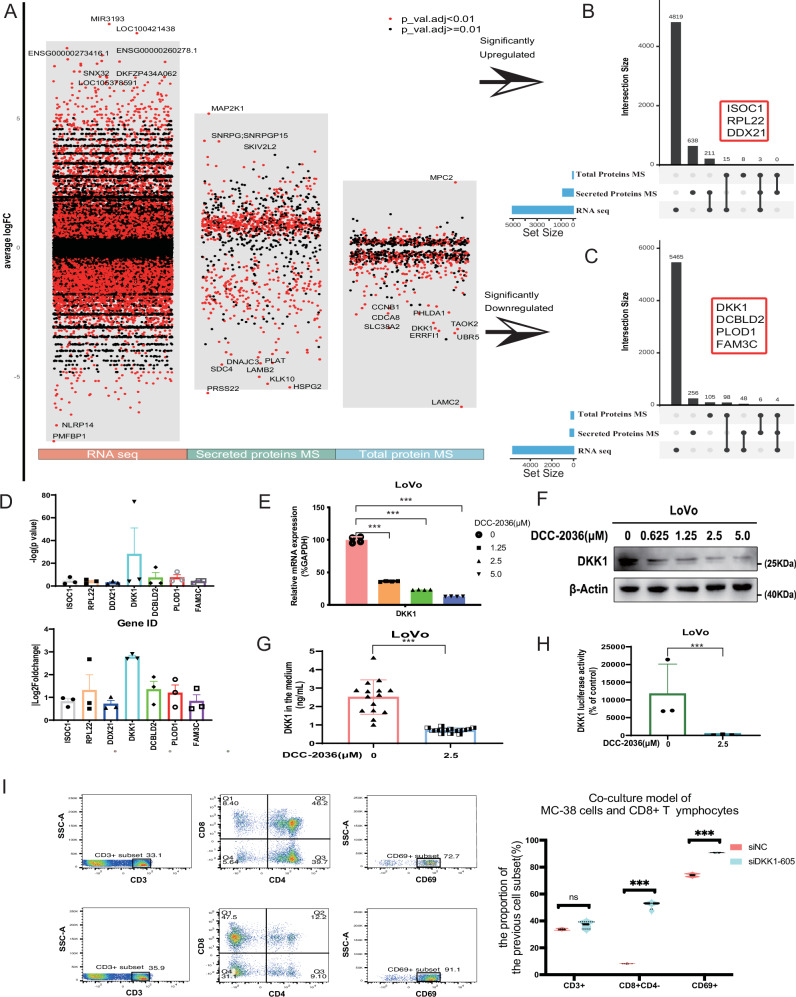
Comprehensive analysis of DCC-2036’s impact on gene and protein expression in LoVo cells. **A** Volcano Plots of Molecular Changes in LoVo Cells: Illustrating alterations in RNA sequencing, total protein, and secreted protein profiles post DCC-2036 exposure (2.5 μM, 24 h). **B**–**C** Upset diagram displaying the intersection of the up- or down-regulated genes/proteins. **D** Scatter Histogram of Gene/Protein Changes: Displaying |log2(Fold Change)| and -log10 (*P* value) for selected genes/proteins. **E**–**H** Analysis of DKK1 in LoVo Cells: Evaluating mRNA levels (q-RT-PCR, E), secreted protein (ELISA, **F**), total protein (Western Blotting, **G**) and promoter activity (Dual-Luciferase Reporter Assay, **H**) post DCC-2036 administration. Data expressed as mean ± SD, analyzed using Student’s t-test (***P < 0.001). **I** Flow cytometric analysis of T cell subsets in the co-culture model of MC-38 cells and CD8^+^ T lymphocytes. The left panels show the gating strategy for CD3^+^, CD4^+^, CD8^+^, and CD69^+^ T cells. The top row represents the siNC (negative control) group, while the bottom row represents the siDKK1-605 group, where DKK1 expression was silenced(left). Quantification of T cell populations including CD3^+^, CD8^+^CD4^-^, and CD69^+^ subsets. The proportion of CD3^+^ cells showed no significant difference between the siNC and siDKK1-605 groups, while CD8^+^CD4^-^ and CD69^+^ populations were significantly increased in the siDKK1-605 group compared to siNC. Data are presented as mean ± standard deviation. Statistical significance was determined using Student’s t-test (**P < 0.001, ns: not significant)(right).

In our study, we analyzed the effects of DCC-2036 on CD8^+^ T cell populations and their functionality within CRC cells using an extensive multi-omics approach, including transcriptomic, proteomic, and secretomic profiling. We treated LoVo cells with DCC-2036 at a 2.5 μM concentration, leading to significant shifts in cellular expression: 4819 genes and 211 proteins were upregulated, along with 638 secreted proteins, while 5465 genes, 105 proteins, and 256 secreted proteins were downregulated (Fig. 2A).

The upset plots revealed co-regulation across various genes and proteins, showing consistent upregulation of ISOC1, RPL22, and DDX21, while DKK1, DCBLD2, PLOD1, and FAM3C were notably downregulated (Fig. 2B-C). Particularly significant was the differential expression of DKK1, which exhibited a Log2(FoldChange) of -2.94 (Fig. 2D). The changes in DKK1 were confirmed by luciferase, qRT-PCR, Western blotting, and ELISA tests, as well as the amounts of mRNA and secreted protein (Fig. 2E-H). We used a co-culture system that included MC-38 CRC cells and CD8^+^ T lymphocytes to determine how DKK1 affects T cell dynamics in the tumor microenvironment (TME). The silencing of DKK1 resulted in discernible alterations in T cell subset distribution relative to the negative control. In the siDKK1-605 group, there was a significant rise in the CD8^+^CD4^+^ T cell population, while in the siNC group, the fraction of CD3^+^ T cells was constant, suggesting that DKK1 silencing had no significant effect on total T cell infiltration. This suggests that DKK1 suppression may facilitate the proliferation or persistence of cytotoxic T cells. Furthermore, CD69^+^ expression, indicative of T cell activation, was markedly elevated in the siDKK1-605 group compared to controls, demonstrating enhanced CD8^+^ T cell activation (Fig. 2I). These results suggest that DKK1 may prevent cytotoxic T cell activation and proliferation in the TME. Hence, it seems that inhibiting DKK1 improves CD8^+^ T cell functioning, which could lead to better anti-tumor immune responses and make DKK1 a suitable target for CRC immunotherapy.

RNA sequencing analysis of MC-38 cells, along with total and CD8^+^ T cells, with and without the presence of DKK1, disclosed substantial enhancement in apoptosis pathways (KEGG pathway: hsa04210), predominantly in CD8^+^ T cells (Fig. S3A). These findings underscore DKK1’s value as a therapeutic target in CRC, influencing survival rates, CD8^+^ T cell infiltration, and immunotherapeutic responses [21, 22].

The CIBERSORT deconvolution analysis also revealed a strong inverse relationship between the abundance of CD8^+^ T cells in the CRC microenvironment and DKK1 expression (Fig. S3B-C). With an area under the receiver operating characteristic curve (AUC ROC) of 0.873, DKK1 successfully distinguished between patients who responded to Anti-PD-1/CTLA-4 therapy and those who did not, suggesting that it may be a useful predictive biomarker for immunotherapy outcomes (Figure S3D). Figure S3E shows that DKK1 expression is inversely related to progression-free survival (PFS) after immunotherapy, suggesting that DKK1 may be an important biomarker for the management of CRC.

### Elucidating FGR’s role as a direct and crucial target for DCC-2036’s efficacy in CRC

**Fig. 3:**
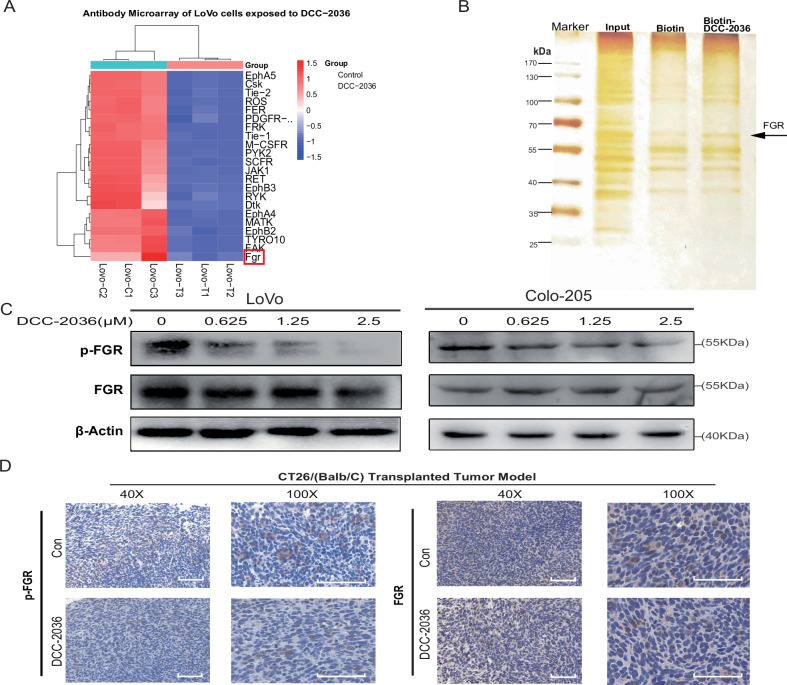
Delineating the role of FGR as a direct target in the efficacy of DCC-2036 in colorectal cancer. **A** Heatmap of Phosphorylated-Tyrosine Protein Kinases in DCC-2036 Treated LoVo Cells: Highlighting significant differences in kinase activity after 24 h exposure to 2.5 μM DCC-2036, as assessed by the PathScan® RTK Signaling Antibody Array Kit (Chemiluminescent Readout). **B** Pull-Down Assay Identifying Direct Targets of DCC-2036: Biotin-tagged DCC-2036 was used to capture interacting proteins in CRC cells, with non-immunoprecipitated LoVo lysates as positive control. **C** Western blot analysis showing the impact of different concentrations of DCC-2036 (0, 0.625, 1.25, and 2.5 μM) on FGR and phosphorylated FGR expression levels in LoVo cells, with β-Actin serving as a loading control. **D** Immunohistochemical analysis of p-FGR and FGR in a CT26 tumor model transplanted into Balb/C mice, comparing treated versus control groups at both 40× and 100× magnification.

Our initial investigations highlighted DKK1 as a likely downstream mediator influenced by DCC-2036 in CRC. With DCC-2036 recognized as a TKI, we pivoted our research to pinpoint the specific kinase upstream of DKK1 impacted by DCC-2036 in CRC cells. Utilizing both a phospho-antibody microarray and a pull-down assay, we noted a marked decrease in the phosphorylation of 11 TKs in LoVo cells treated with DCC-2036, demonstrating the inhibitor’s potent activity (Fig. 3A). Employing biotin-labeled DCC-2036 in the pull-down assay helped identify FGR as a prominent interacting protein (Fig. 3B). Further dose-response analysis showed a reduction in phosphorylated FGR (Y416) levels, while total FGR remained unchanged (Fig. 3C). Immunohistochemical assessments of CT-26 tumor models treated with DCC-2036 confirmed the suppression of p-FGR, emphasizing FGR’s critical role in the drug’s action mechanism (Fig. 3D, Fig. S4).

**Fig. 4:**
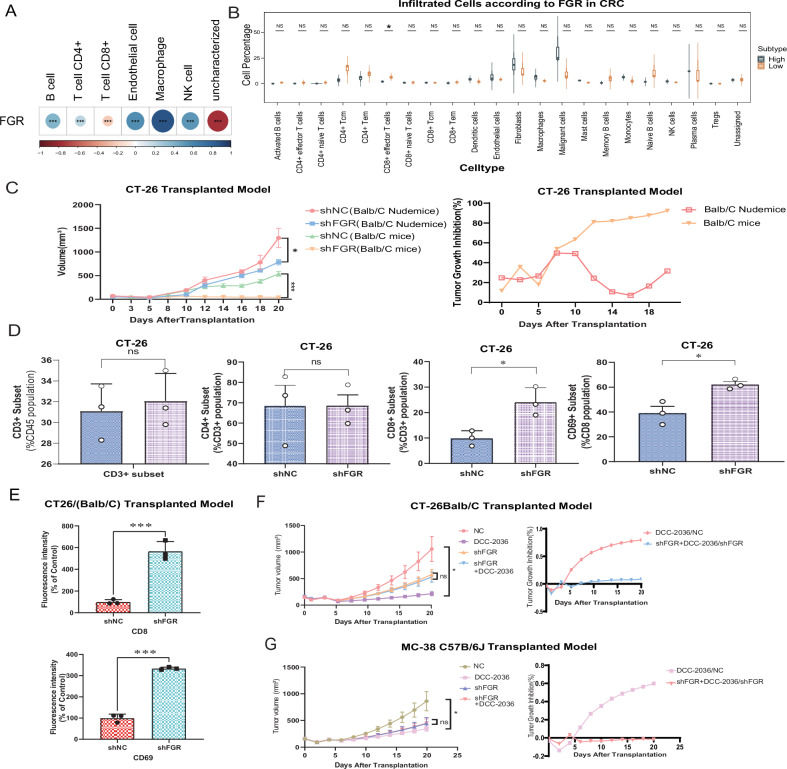
Delineating the role of FGR as a critical target in the efficacy of DCC-2036 in colorectal cancer. **A** Correlation Analysis Between FGR and Major Immune Cells in CRC TME: Illustrates the correlation between FGR expression and seven major immune cells in the TME of CRC. Positive correlations are shown in blue shade, while negative correlations are in red. The size of each node reflects the magnitude of the correlation. Analysis performed using Pearson’s correlation, two-sided, (***P < 0.001). **B** Boxplot Graph of Cell Infiltrates in CRC Samples with Varied FGR Expression: Demonstrates differences in cell infiltrates between three paired CRC samples, categorized by low and overexpressed FGR levels. Data sourced from Single Cell Expression Atlas-EBI (E-MTAB-8410) titled “Single-cell sequencing of colorectal tumors and adjacent non-malignant colon tissue”. Statistical significance was assessed using Student’s t-test (*, *P* <0.05). **C** Analysis of CT-26 Homografts: Depicts growth and inhibitory curves of CT-26 homografts generated via intratumor injection of shFGR/shNC lentiviruses. CT-26 cells were subcutaneously administered to 6-week-old Balb/C nude mice and Balb/C mice, with each group consisting of *n* = 8 mice, data expressed as mean ± SD, analyzed using Student’s t-test (*P < 0.05,***P < 0.001). **D** Lymphocyte Subpopulation Percentages: Shows the percentage of CD3, CD4, CD8, and CD69 lymphocyte subpopulations from three independent experiments, the data was reported as the mean ± standard deviation and subjected to statistical analysis using Student’s t-test (*P < 0.05, ns = not significant). **E** Immunofluorescent Staining in Implanted Tumors of Balb/C mice: The percentage of fluorescence intensity of CD8 and CD69 from three independent experiments. The data was presented as the mean ± standard deviation and analyzed utilizing Student’s t-test, with statistical significance denoted by ***P < 0.001. **F** CT-26 shFGR/shNC Homograft Mouse Models Treated with DCC-2036: The left panel shows changes in tumor volume over time. Right panel depicts the tumor growth inhibition index at harvest, represented by curves. **G** MC-38 shFGR/shNC Homograft Mouse Models Treated with DCC-2036: the left panel illustrating the changes in tumor volume over time, and the right panel presenting the tumor growth inhibition index at harvest. CT-26/MC-38 cells, either shFGR or shNC, were subcutaneously injected into mice. These mice were then orally treated with DCC-2036 (50 mg/kg) once every two days. Group size: n = 8. Statistical analysis was performed using Student’s t-test for individual comparisons (*p < 0.05, **p < 0.01). Data represent mean ± SD from experiments conducted in triplicate.

There was a strong correlation between FGR expression and immune cell infiltration in COAD samples, according to correlation studies conducted on the TCGA dataset (Fig. 4A, *P* < 0.05). Examination of single-cell RNA sequencing data (E-MTAB-8410) highlighted CD8^+^ effector T cells as the subtype most substantially reduced in CRC samples with high FGR expression (Fig. 4B, *P* < 0.05). To study the immunomodulatory function of FGR, researchers used CT-26 models to conduct deletion tests, which revealed a significant reduction in tumor growth in immunocompetent (Balb/C) mice (Fig. 4C, Fig. S5A). Flow cytometry results confirmed that FGR knockdown mimicked DCC-2036’s effects, notably enhancing CD8^+^ T cell activation and numbers (Fig. 4D, Fig. S5B). Immunofluorescence staining for CD8 and CD69 supported these observations (Fig. 4E, Fig. S5C). Additionally, FGR knockdown using shFGR lentiviruses in CT-26 and MC-38 subcutaneous tumor models led to reduced tumor volumes, although FGR-deficient groups showed lessened sensitivity to DCC-2036 (Fig. 4F-G, Fig. S5D-E). These results demonstrate that FGR is an essential target of DCC-2036’s action mechanism in CRC cells.

### Suppression of DKK1 by DCC-2036 through FGR targeting in colorectal carcinoma

**Fig. 5:**
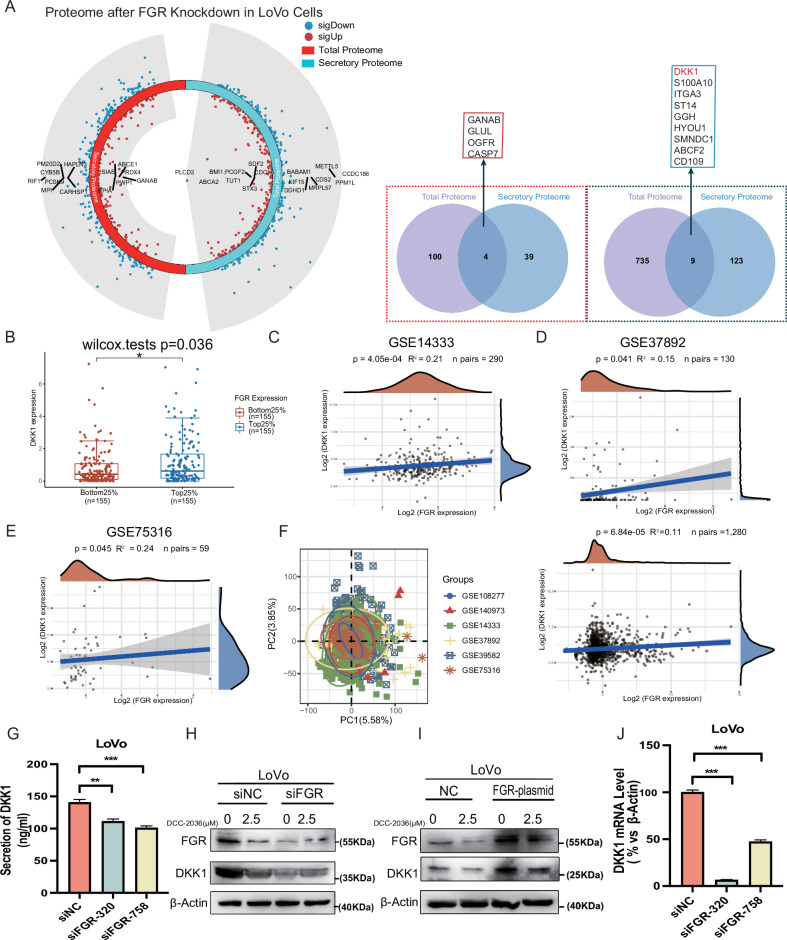
DCC-2036 mediated regulation of DKK1 through targeting FGR in colorectal cancer. **A** Volcano Plots of Molecular Changes in LoVo Cells: Illustrating alterations in RNA sequencing, total protein, and secreted protein profiles post DCC-2036 exposure (2.5 μM) and FGR silencing(siFGR). **B** A comparative analysis was conducted to examine the expression of DKK1 in CRC tumors from the TCGA database, with a focus on the levels of FGR expression. The tumor samples were categorized into two groups based on FGR expression levels: ≤25% indicating low expression and ≥25% indicating overexpression. A scatterplot was used to compare the levels of DKK1 expression in samples falling within the highest (top 25%) and lowest (bottom 25%) quartiles of FGR expression. Error bars represent standard deviation (SD). Statistical significance between groups was determined using Student’s t-test (*P < 0.05). **C–F** The present study employed Spearman’s correlation analysis to evaluate the association between FGR and DKK1 gene expression levels, utilizing data sourced from the GEO database (www.ncbi.nlm.nih.gov/geo). **G** FGR knockdown resulted in a marked decrease in DKK1 expression, as evidenced in secretome profiles ELISA assays. Statistical analysis was performed using Student’s t-test for individual comparisons (**P < 0.01,***P < 0.001). Data represent mean ± SD from experiments conducted in triplicate. **H** The expression of DKK1 in LoVo cells treated with the combinations of FGR silenced and DCC-2036 (0 and 2.5 μM) was determined through Western blotting. **I** The expression of DKK1 in LoVo cells treated with the combinations of overexpression FGR and DCC-2036 (0 and 2.5 μM) was determined through Western blotting. **(** DKK1 mRNA level was measured by q RT‐PCR after shFGR treatment. Statistical analysis was performed using Student’s t-test for individual comparisons (***P < 0.001). Data represent mean ± SD from experiments conducted in triplicate.

Our investigation identified DKK1 as a target significantly downregulated by DCC-2036 in colorectal carcinoma (Fig. 5A). To uncover how DCC-2036 modulates DKK1 expression via FGR, we examined the association between FGR and DKK1 levels. The results revealed that increased FGR expression correlates with higher DKK1 levels, indicating a regulatory interaction between these proteins (Fig. 5B-F).

FGR knockdown led to a marked decrease in DKK1 expression, confirmed by ELISA assays evaluating secretome profiles (Fig. 5G). Intriguingly, post-FGR silencing, DCC-2036 did not further reduce DKK1 levels (Fig. 5H), and FGR overexpression countered the DCC-2036-induced suppression of DKK1 (Fig. 5I). Quantitative RT-PCR analysis further supported these findings by showing reduced DKK1 mRNA levels following FGR knockdown (Fig. 5J). These observations suggest that DCC-2036 suppresses DKK1 predominantly through targeting FGR, which leads to the transcriptional downregulation of DKK1.

### Regulation of DKK1 by FGR through PI3K-AKT-mediated SP1 binding to the DKK1 promoter in CRC

**Fig. 6:**
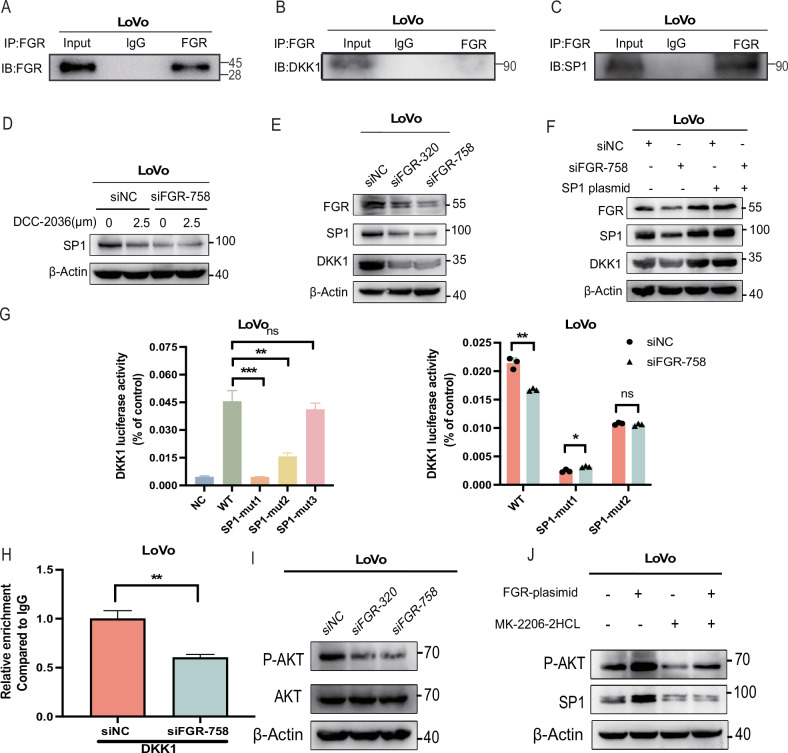
FGR regulates DKK1 through PI3K-AKT-mediated binding of SP1 to the DKK1 promoter in colorectal cancer. **A** Immunoprecipitation (IP) and immunoblotting (IB) analysis demonstrating that FGR protein is pulled down using an anti-FGR antibody in LoVo cells. FGR was effectively immunoprecipitated, confirmed by immunoblotting for FGR. **B** Co-immunoprecipitation of DKK1 with FGR. The IP of FGR was performed, followed by IB for DKK1, indicating an interaction between FGR and DKK1. **C** Co-immunoprecipitation of SP1 with FGR in LoVo cells. After FGR was immunoprecipitated, the presence of SP1 was detected by immunoblotting**. D** The expression of SP1 in LoVo cells treated with the combinations of FGR silenced and DCC-2036 (0 and 2.5 μM) was determined through Western blotting. **E** FGR was knockdown via shFGR lentivirus in LoVo cells, and the protein levels of SP1 and DKK1 were determined by WB. **F** The expression of DKK1 and SP1 in LoVo cells treated with the combinations of SP1 overexpression and DCC-2036 (0 and 2.5 μM) was determined through Western blotting. **G** The left image is transfected with either the control vector alone or with the vector containing promoter fragments (mut1, mut2, mut3) in LoVo cells. Luciferase reporter activity was measured 48 hours post-transfection using a dual-luciferase assay. The right image is the effect of shFGR on the DKK1 promoter activity, which measured by luciferase reporter assay. The data was reported in terms of the mean ± standard deviation and subjected to analysis using Student’s t-test, with statistical significance indicated by ns = not significant, *P < 0.05, **P < 0.01, and ***P < 0.001. Scale bar = 2.0 cm. **H** ChIP assays were employed to show the direct binding of SP1 to DKK1 promoter regions (−167bp to −159bp and −118bp to −108bp) after shFGR treatment. The data was presented as mean ± standard deviation and analyzed using Student’s t-test, with statistical significance indicated by **P < 0.01. **I** The P-AKT and AKT protein levels were determined by WB after shFGR treatment. **J** the SP1 and DKK1 protein levels were determined by WB after AKT inhibitor treatment (MK-2206-2HCL) and overexpression of FGR (right).

To elucidate the role of the tyrosine kinase FGR in regulating DKK1 transcription within CRC cells, we initially conducted a co-immunoprecipitation assay. Figure 6A demonstrates successful FGR immunoprecipitation using an anti-FGR antibody, verifying the specificity of this technique. Further analysis to determine if FGR directly associates with DKK1 involved immunoprecipitation using an anti-FGR antibody followed by immunoblotting with an anti-DKK1 antibody. These results showed no direct interaction between FGR and DKK1 in LoVo cells (Fig. 6B), prompting us to investigate indirect pathways through which FGR might influence DKK1 transcription. This phase involved FGR co-immunoprecipitation paired with mass spectrometry and the use of predictive online tools to identify possible transcription factors impacting DKK1 regulation. This approach pinpointed SP1 as a key mediator through which FGR regulates DKK1 transcription, supported by Venn network analysis (Fig. S6A).

Subsequent experiments confirmed the association between FGR and SP1 via immunoprecipitation (Fig. 6C). To understand how DCC-2036 impacts SP1 expression through FGR, we observed reductions in SP1 expression with both DCC-2036 treatment and FGR siRNA. Following FGR knockdown, DCC-2036 did not further decrease SP1 levels (Fig. 6D), indicating that DCC-2036’s suppression of SP1 primarily occurs through FGR inhibition. The silencing of FGR in LoVo cells resulted in decreased levels of both DKK1 and SP1 (Fig. 6E), whereas SP1 overexpression reversed the suppressive effects on DKK1 and SP1 levels caused by siFGR (Fig. 6F). These outcomes demonstrate that FGR controls DKK1 expression via the transcription factor SP1, highlighting a complex regulatory mechanism within the cellular milieu.

To elucidate the specific locations of SP1’s regulatory action within the DKK1 promoter, we utilized the JASPAR database to predict SP1 binding sites. Mutations were then introduced at these predicted sites, followed by luciferase assays to assess their effects. Mutations at sites 1 and 2 significantly decreased promoter activity, effectively mitigating the transcriptional repression caused by siFGR (Fig. 6G). Chromatin immunoprecipitation (ChIP) assays post-shFGR treatment targeted these regions, specifically −118bp to −108bp and −167bp to −159bp, highlighting the critical roles these sequences play in the interaction between SP1 and DKK1 (Fig. S6B).

Further investigation into SP1 modulation by FGR revealed its influence on PI3K-AKT signaling, a pathway where AKT directly affects SP1’s expression and activity [23, 24]. Using an AKT inhibitor (MK-2206 2HCL) reversed the increase of SP1 expression induced by FGR, and a significant decrease in AKT phosphorylation was noted after FGR silencing (Fig. 6I). These data provide more evidence that the PI3K-AKT pathway is the mediator of FGR’s effect on SP1 (Fig. 6J).

In conclusion, our study provides a detailed exploration of the indirect mechanism by which FGR influences DKK1 transcription in CRC via the PI3K-AKT pathway, facilitating SP1’s interaction with the DKK1 promoter [25]. This insight enhances our understanding of the molecular dynamics in CRC and opens avenues for targeted therapeutic strategies in precision medicine.

### Correlative analysis of DKK1, SP1, FGR, and p-FGR in CRC: prognostic implications

**Fig. 7:**
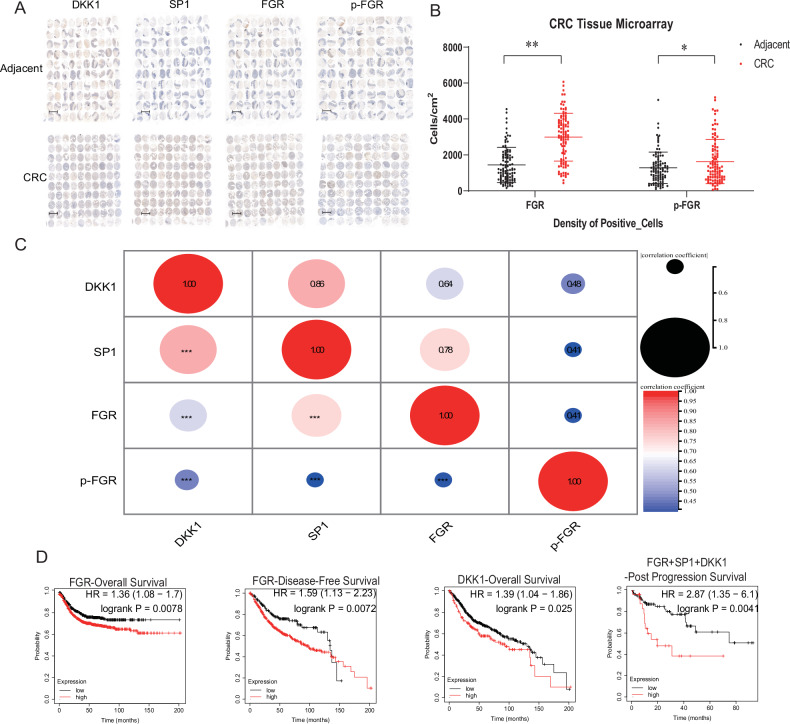
Correlation of SP1, DKK1, FGR, p-FGR expression with clinical outcomes in CRC. **A** TMA Staining Interpretation: Immunohistochemical staining of DKK1, SP1, FGR, and phosphorylated FGR (p-FGR) in adjacent and cancerous CRC tissues. The staining was interpreted by the pathology department of the first affiliated hospital of the University of South China. Scale bars = 50 μm. **B** Boxplot Analysis of Protein Expression: The density of positive cells for FGR and p-FGR in adjacent and cancerous CRC tissues is shown. Statistical significance was assessed using Student’s t-test (*P < 0.05, **P < 0.01). **C** Protein Expression Correlation Matrix and Scatter Plot: This section displays the correlations among the protein expressions of DKK1, SP1, FGR, and p-FGR in CRC. The matrix diagram and dotplot/correlation are provided to illustrate these relationships. Statistical significance was assessed using Student’s t-test (***P < 0.001). **D** Kaplan-Meier Survival Analysis: The Kaplan-Meier survival curves for FGR, DKK1, and the mean of FGR, DKK1, and SP1 are presented. The curves were generated using the Kaplan Meier plotter to assess the association between protein expression levels and patient survival.

Our research utilized immunohistochemical techniques on CRC tissue microarrays to examine the associations among DKK1, SP1, FGR, and phosphorylated FGR (p-FGR) protein levels (Fig. 7A). The analysis highlighted a notable overexpression of both FGR and p-FGR in CRC samples (Fig. 7B), with these proteins showing significant positive interactions and correlations with each other (Fig. 7C).

Survival analyses were subsequently carried out to explore the clinical repercussions of these findings. The data disclosed a correlation between the overexpression of FGR and reduced overall survival (OS) with a hazard ratio (HR) of 1.36 (95% CI: 1.08 – 1.7, log-rank *P* = 0.0078) and DFS with an HR of 1.59 (95% CI: 1.13–2.23, log-rank *P* = 0.0072) in CRC patients. Likewise, elevated levels of DKK1 were linked to decreased OS (HR = 1.39, 95% CI: 1.04–1.86, log-rank *P* = 0.025). Additional analysis demonstrated that average expression levels of SP1, DKK1, and FGR significantly predicted post-progression survival in CRC, with an HR of 2.87 (95% CI: 1.35–6.1, log-rank *P* = 0.0041) (Fig. 7D). These results underscore the potential of these markers as predictors for prognosis and survival in CRC, suggesting their role in tailored therapeutic strategies.

### Influence of FGR on immune checkpoint inhibitor efficacy in CRC

Immunotherapy is increasingly recognized for its potential in treating CRC due to ongoing investigations into the disease’s pathogenesis. However, immune checkpoint inhibitors (ICIs) are not as effective in CRC because CD8^+^ T lymphocytes in the TME are functionally impaired and few [26].

**Fig. 8:**
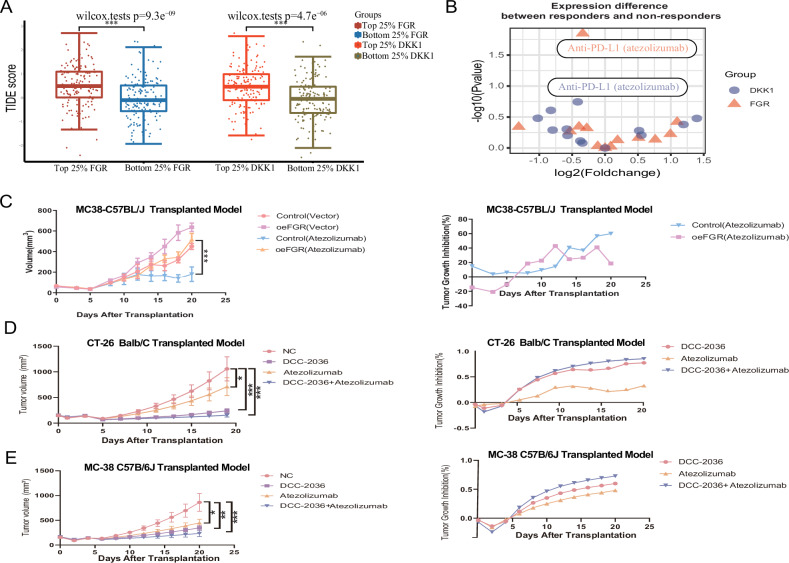
Modulation of Immune Checkpoint Inhibitor Sensitivity by FGR in Colorectal Cancer. **A** TIDE Prediction Score Analysis. This scatterplot summarizes the differences in TIDE prediction scores between samples with high (top 25%) and low (bottom 25%) expressions of FGR/DKK1. Error bars represent the standard deviation (SD). Statistical significance was assessed using Student’s t-test (*** *P* < 0.001). **B** Transcriptomic and Genomic Analyses of FGR/DKK1: These analyses were conducted on pre-treated neoplastic biopsies from patients responsive and resistant to immunotherapy (such as anti-PD-L1 and anti-PD1 therapies), utilizing the TISIDB database. **C** Growth curves for MC-38 homografts stably transfected with overexpression FGR lentivirus or control lentivirus, treated with Atezolizumab. MC-38/oeFGR and MC-38/Control cells were subcutaneously injected into congenic mice, which were then orally treated with DCC-2036 as previously described. Group size: *n* = 4. **D** Growth curves of implanted CT-26 subcutaneous tumors in Mice models treated with combinations of DCC-2036 and Atezolizumab or separated in 6–8 week old Balb/C mice. **E** Similar to part, growth curves of implanted MC-38 subcutaneous tumors in Mice models treated with combinations of DCC-2036 and Atezolizumab or separated in 6–8 week old C57BL/6 J mice. Oral administration of DCC-2036 was at 50 mg/kg every other day, and Atezolizumab was injected at 5.0 mg/kg twice a week. Data are from three biological replicates, analyzed using Student’s unpaired t-test (*P < 0.05, **P < 0.01, ***P < 0.001).

A strong predictor of patient response to ICIs is the T-cell dysfunction and exclusion (TIDE) model, which assesses tumor-infiltrating lymphocytes (TILs). Analysis of TCGA indicated significantly higher TIDE prediction scores in CRC samples with the top 25% expressions of FGR or DKK1 compared to those in the bottom 25%, implying a diminished response to immunotherapy in these higher expressing groups (Fig. 8A) [27]. Atezolizumab is a PD-L1 targeting antibody; nevertheless, it is associated with reduced efficacy when FGR and DKK1 levels are high, according to TISIDB, a database that documents tumor-immune interactions (Fig. 8B) [28–30]. By enhancing T cell activation and infiltration into the TME, atezolizumab is known to improve T cell cytotoxicity. In the MC-38 tumor-bearing mouse model, FGR overexpression negated the suppressive impact of atezolizumab (Fig. 8C, Fig. S7A), supporting the notion that CD8^+^ T cell deficits within the tumor milieu limit immunotherapeutic outcomes in CRC.

Given this backdrop, the suppression of FGR could potentially heighten CRC’s responsiveness to ICI therapy. DCC-2036, an FGR inhibitor, may therefore augment the therapeutic effects of atezolizumab. Comparative studies in CT-26 and MC-38 homograft models demonstrated that combining DCC-2036 with atezolizumab markedly reduced CRC progression more effectively than either treatment alone (Fig. 8D-E, Figure S7B-C). These insights suggest a strategic avenue to enhance ICI therapy’s impact in CRC by targeting FGR modulation.

## Discussion

Recent studies have heightened interest in how TKIs modify host immunity, particularly within the TME. For instance, EGFR-specific TKIs have been found to trigger a comprehensive interferon response in cancer cells, thereby affecting the mobilization and participation of immune cells in the treatment response [31]. Similarly, SAR131675, an inhibitor of VEGFR3, changes the makeup of immune cells within the TME, increasing CD4^+^ T cells and decreasing the F4/80^+^ Ly6Clow subset [32]. Conversely, the effects of DCC-2036, a central focus of our study, on the TME in CRC are still being delineated. Previous research suggests that DCC-2036 impedes tumor growth and metastasis in breast cancer by targeting Tie-2^+^ macrophages [16], yet its specific impact on CRC’s TME requires further exploration.

Our current research elucidates the inhibitory effects of DCC-2036 on CRC, observing varied responses in in vivo models using both immunocompetent and immunodeficient mice. In nude mice, DCC-2036 demonstrated moderate CRC suppression; however, its efficacy was more pronounced in immunocompetent mice, highlighting its significant immunomodulatory effects. Notably, after being treated with DCC-2036, Balb/C mice showed a significant decrease in the size of their CRC tumors. It is possible that the distinct anticancer effects of DCC-2036, especially in relation to T-cell-mediated immunoregulation, are explained by the T-cell deficit in Balb/C nude mice as opposed to Balb/C animals. Through flow cytometry, immunohistochemistry, and immunofluorescence assays, we confirmed that DCC-2036 enhances both the infiltration and functionality of CD8^+^ T cells in CRC.

Tumors with high levels of tumor cell infiltration are called hot tumors, whereas tumors with low levels of tumor cell infiltration are called cold tumors. Mature T lymphocytes are normally grouped into CD8^+^ cytotoxic T cells and CD4+ helper cells. TILs, especially CD8^+^ T cells [33], have been associated with improved prognosis in hepatocellular carcinoma [34]. Contrary to earlier studies [35], DCC-2036 has demonstrated a significant increase in the recruitment and activation of cytotoxic T cells (CD8^+^CD4^-^ and CD8^+^CD4^-^CD69^+^), without affecting the populations of CD4^+^ CD8^-^ and CD4^+^ CD8^-^CD69^+^ cells. This demonstrates how DCC-2036 stands out from other TKIs when it comes to boosting anti-tumor CD8^+^ T cell responses.

The primary target of DCC-2036 was identified as FGR kinase through tyrosine kinase antibody and pull-down assays. Knockdown of FGR in CT-26 and MC-38 subcutaneous tumor models resulted in reduced tumor volumes, although these FGR-deficient groups showed less responsiveness to DCC-2036. This indicates that FGR is crucial for mediating DCC-2036’s therapeutic efficacy in CRC, emphasizing its value as a therapeutic target.

Our research contributes to the understanding of CRC treatment by demonstrating DCC-2036’s effectiveness in tumors expressing FGR. Using shFGR lentiviruses, we achieved FGR knockdown in CT-26 and MC-38 homograft mouse models, leading to significant tumor reduction. This confirms the importance of FGR in the therapeutic mechanism of DCC-2036 and highlights the precision of lentiviral technology in gene regulation. The reduced responsiveness in FGR-deficient tumors underscores FGR’s essential role in enhancing DCC-2036’s effectiveness, suggesting that FGR expression could serve as a prognostic marker for treatment outcomes. These results underline the significance of molecular profiling in tailoring CRC therapies and advocate for further research into combination treatments to maximize DCC-2036’s benefits. This study demonstrates the critical role of genetic engineering in advancing personalized cancer treatments, significantly enriching the oncology field.

FGR, belonging to the Src family of kinases, can autophosphorylate at Y416 and dephosphorylate at Y527, activating subsequent signaling pathways [36]. Previous studies have highlighted FGR’s potential role in the progression of ovarian carcinoma and its emerging clinical relevance in CRC [37]. It has been observed that CRC cases with active FGR frequently exhibit advanced TNM stages, poor differentiation, and limited necrosis [37]. This research contributes further insights into FGR’s immunomodulatory roles in CRC. The silencing of FGR markedly boosted CD8^+^ T cell infiltration and activation, significantly curtailing tumor growth in vivo. Notably, in immunocompetent mice, tumors with FGR knockdown did not grow beyond 50 mm^3^, whereas no significant growth difference was observed in immunodeficient mice. Based on these results, FGR targeting may be a good strategy for CRC immunotherapy.

The presence of tumor-infiltrating CD8^+^ T cells is crucial for effective CRC immunotherapy. Our analysis uncovered a negative correlation between FGR expression and response to atezolizumab using the TISIDB database [27]. Furthermore, an increase in FGR expression mitigated the inhibitory effects of atezolizumab in a CRC animal model. The combined administration of atezolizumab and DCC-2036 showed enhanced sensitivity to atezolizumab’s effects, suggesting that the synergistic potential of combining DCC-2036 with other immunotherapies deserves further exploration.

Distinct alterations in DKK1 expression post-DCC-2036 treatment were revealed through comprehensive multi-omics analyses. Elevated DKK1 levels in CRC are related to a worse prognosis, more advanced disease stages, and metastasis [38–40]. Originally identified as a protein involved in embryonic development, DKK1 is now thought to play a role in cancer progression by creating an environment where cancer cells can thrive and multiply.

DKK1 inhibits β-catenin activation, thereby suppressing the Wnt signaling pathway, which is essential for tumor suppression. An immunosuppressive TME is linked to DKK1 overexpression in CRC, making it harder for the immune system to mount a successful anti-tumor response. The recruitment of immunosuppressive cells such as myeloid-derived suppressor cells and M2-polarized macrophages is the main mechanism by which increased DKK1 levels are associated with reduced CD8^+^ T cell infiltration and activity inside the TME [41, 42]. The role of DKK1 in fostering immunosuppression is partly facilitated through its interaction with CKAP4 on macrophages, activating the PI3K-Akt pathway and promoting an M2 phenotype that suppresses anti-tumor immunity. Additionally, DKK1 directly impedes T cell receptor signaling, reducing CD8^+^ T cell responsiveness to tumor antigens and contributing to resistance against therapies like anti-PD-1 checkpoint blockade in CRC [43, 44].

Our research indicates that DCC-2036 specifically reduces the transcription levels of DKK1. Mass spectrometry revealed that SP1, a transcription factor involved in regulating DKK1 in CRC, interacts with FGR. This interaction suggests a mechanism through which FGR may facilitate the tyrosine phosphorylation of transcription factors, including TBK1 and possibly SP1, thereby promoting DKK1 transcription [45, 46]. These insights contribute to a deeper understanding of DKK1’s regulatory complexity and its impact on CRC progression.

The immunosuppressive characteristics of DKK1 play a significant role in shaping the tumor immune microenvironment by reducing the proliferation and functionality of cytotoxic CD8^+^ T cells, which are essential for effective anti-tumor responses. Furthermore, elevated levels of DKK1 are associated with heightened expression of PD-L1, diminished T cell infiltration, and increased immune evasion, all of which play a role in the advancement and resistance to therapy in CRC [44].

Given these findings, DKK1 emerges as a valuable therapeutic target in CRC. Inhibiting DKK1 could rejuvenate CD8^+^ T cell functionality, reorient the TIME towards an anti-tumor stance, and potentially augment the effectiveness of immunotherapies, including checkpoint inhibitors. Ongoing research explores combining DKK1 inhibitors with ICB to counteract resistance and improve outcomes for CRC patients. Therefore, targeting DKK1 may be pivotal in boosting the efficacy of cancer immunotherapy and enhancing CRC patient prognosis.

Earlier studies have demonstrated FGR’s role in modulating PI3K-AKT signaling. This research identifies that AKT phosphorylation of SP1 at specific residues (S42, T679, and S698) enhances SP1’s stability and DNA-binding capabilities, substantially increasing its functional impact and resulting in heightened expression of target genes. These insights reveal a fundamental regulatory mechanism in gene transcription [45, 47]. Furthermore, SP1 is shown to escalate DKK1 expression by intensifying its promoter activity in a concentration-dependent manner, highlighting SP1’s essential role in gene regulation dynamics [25].

Our study unveils a complex regulatory mechanism in CRC, showing that FGR modulates DKK1 transcription via the PI3K-AKT pathway, with SP1 acting as a critical intermediary. This indirect modulation pathway, which departs from anticipated direct interactions, provides new insights into CRC’s molecular dynamics and potential therapeutic targets. By emphasizing SP1’s pivotal role in the FGR-DKK1 axis and detailing its regulation through PI3K-AKT signaling, our research enhances the understanding of the elaborate signaling networks that govern gene expression in cancer.

Our investigation explores unconventional routes, focusing on the impact of tyrosine kinase activity on transcription factors and gene expression in CRC. This approach offers fresh perspectives on the molecular mechanisms of the disease. The finding that FGR’s influence on DKK1 is mediated through a signaling pathway involving SP1 highlights the intricate regulatory processes that dictate cancer cell behavior and identifies the FGR-PI3K-AKT-SP1 pathway as a valuable target for therapeutic intervention.

This research establishes a link between the expression levels of FGR, DKK1, SP1, and patient outcomes in CRC, underlining the potential use of these molecular markers in prognostication and treatment planning. Specifically, their relationship with the responsiveness to ICIs, including the effects on atezolizumab efficacy, suggests innovative methods to enhance immunotherapy outcomes by targeting FGR.

The intricate nature of the PI3K-AKT signaling pathway, marked by its various downstream effectors and feedback loops, poses challenges in comprehensively understanding the impact of FGR on SP1 and DKK1 expression. Additionally, translating these findings from experimental models to clinical applications requires careful consideration given the diverse nature of human cancers. The findings indicate a significant role of the FGR-PI3K-AKT-SP1-DKK1 axis in CRC; however, further investigation is essential to understand the broader effects of targeting this pathway in various cancer types and among different patient populations. This additional examination is essential for assessing the viability of this axis as a therapeutic target in cancer treatment.

In summary, our research highlights the potential effectiveness of DCC-2036 in managing CRC, particularly through its immunomodulatory properties, marking a significant step forward in therapeutic strategies. The promising aspects of DCC-2036 necessitate further exploration of its direct anti-tumor activities and its role within the FGR signaling pathway. Such detailed analysis is crucial for advancing future research, deepening our understanding of CRC pathophysiology, and developing more precise and potent treatments. Additionally, our study contributes to the field of CRC by detailing the pivotal role of the FGR-PI3K-AKT-SP1 pathway in regulating DKK1 expression, thus broadening our knowledge of the molecular underpinnings of CRC and suggesting new directions for innovative therapeutic approaches, especially in improving the effectiveness of immunotherapies.

The need for ongoing studies to explore the therapeutic potential and limitations of targeting the FGR-PI3K-AKT-SP1-DKK1 axis extends beyond CRC, encompassing a wider range of cancers. This thorough approach highlights the importance of sustained and comprehensive research to exploit complex molecular interactions fully, potentially transforming the approach to cancer therapy.

## Conclusion

The anti-tumor effects of DCC-2036 on CRC are mediated by its capacity to activate anti-tumor T-cell immunity via the FGR/AKT/SP1/DKK1 axis. The results present strong support for the effectiveness of DCC-2036 as a treatment for CRC, highlighting its possible relevance in a wider range of cancer therapies.

## Methods

### Cell culture

The Chinese Academy of Sciences’ Cell Bank was utilized to obtain the CRC cell lines LoVo and Colo-205. The cell lines CT-26 and MC-38 were from Shanghai Zhongqiao Xinzhou Biotechnology Co., Ltd. The LoVo cells were grown in Gibco’s F-12 Nutrient Mix medium (#11765054), whilst the Colo-205, MC-38, and CT-26 cells were kept in Gibco’s RPMI-1640 medium (#11875093). In order to enhance the media, 10% fetal bovine serum (FBS) from Biological Industries in Northern Kibbutz Beit Haemek, Israel, and 1% penicillin/streptomycin from Gibco (#10378016) were added. The cell cultures were kept in an incubator with 5% CO2 at 37 °C.

### Construction of plasmids, small interfering RNA (siRNA), short hairpin RNA (shRNA), and viral infection

FGR-targeting plasmids and siRNAs were procured from Suzhou GenePharma Gene Co., Ltd. (Suzhou, China). shRNAs were obtained from Shanghai Jikai Gene Chemical Technology Co., Ltd. (Shanghai, China). The sequences for the shRNAs, siRNAs, and plasmids utilized are detailed in Supplementary Table 1.

### Chemicals and antibodies

DCC-2036, was procured from Selleck in Houston, TX, USA. We dissolved it in DMSO and kept it at -20°C for our in vitro investigations. A solution of DCC-2036 in a 0.5% carboxymethylcellulose/1% Tween 80 was given orally to animals at a dosage of 50 mg/kg/day for the in vivo trials. The following antibodies were employed: phospho-FGR (Y412) (Cell Signaling Technology #5724), FGR (Cell Signaling Technology, #8198), β-actin (Cell Signaling Technology, #4970), DKK1 (Proteintech, #21112-1-AP), SP1 (Proteintech, #21962-1-AP), and horseradish peroxidase (HRP)-conjugated secondary antibodies (Merck Millipore, #AP-132P, Darmstadt, Germany). For flow cytometry, the antibodies used were: fixable viability stain 780 (BD Horizon™, #565388), CD45 (BD Horizon™, #550994), CD3 (Invitrogen, #11-0031-82), CD8 (Invitrogen, #25-0081-81), CD4 (BD Horizon™, #553051), CD69 (Invitrogen, #12-0691-81), and purified rat anti-mouse CD16/CD32 (Mouse Fc Block™, BD Horizon™, #553141). For immunohistochemical (IHC) staining, antibodies included p-FGR(Invitrogen, #PA5-105883), FGR(Affinity, #DF6803), IFN-γ (Affinity, #DF6045), IFN-α (Proteintech, #18013-1-AP), DKK1(Proteintech, #21112-1-AP), SP1 (Proteintech, #21962-1-AP), CD8 (Affinity, #AF5126), and CD69 (CST, #5724). For immunofluorescence (IF) staining, utilized were CD3 (Santa Cruz Biotechnology, #sc-59013), CD4 (Novus, #NBP1-19371), CD8 (Novus, #NBP2-29475), and CD69 (Santa Cruz Biotechnology, #sc-373799). Anti-mouse IgG (H + L) F(ab’) fragment (Alexa Fluor® 488 Conjugate, CST, #4408S) and anti-rabbit IgG (H + L) F(ab’) fragment (Alexa Fluor® 594 Conjugate, CST, #8889S) were also used. For MC-38 CRC cells and CD8^+^ T lymphocytes co-culture system, antibodies included anti-mouse CD3 antibody (BD Pharmingen, #553057), CD28 antibody (#553294, BD Pharmingen).

### Animals

The Slyke Jingda Laboratory in Changsha, China, provided the female BALB/c, C57BL/6, and BALB/c nude mice, which were aged 6-8 weeks and kept in an environment free of pathogens.

For colon carcinoma modeling, four models were utilized. For subcutaneous transplantation, FGR-KO and FGR-OE CT26 cells (2.5 × 10^5^) or MC-38 (2.5 × 10^5^) were grafted subcutaneously in the right/left flank of BALB/c mice (CT-26), C57BL/6 mice (MC-38) or BALB/c Nude mice (CT-26, MC-38). Using the formula a^2^×b×0.5, where an is the minor diameter and b is the diameter perpendicular to a, tumor volumes were measured every other day. To reduce potential bias, tumor volume assessments were performed by an investigator blinded to the experimental group assignments. Prompt xenograft extraction, weighing, storage, and fixation followed euthanasia. The NIH Guide for the Care and Use of Laboratory Animals was followed throughout the course of this research. Sodium pentobarbital anesthesia (3.5% [w/v], 1 ml/kg) was one of the methods used to lessen the pain that animals endured during surgeries. The University of South China’s ethics committee gave their stamp of approval to the study’s procedures.

$$\% {\rm{TGI}}=\left[1-\frac{({\rm{Vtreat}}-{\rm{Vtreat}}0)/{\rm{Vtreat}}0}{({\rm{Vcontrol}}-{\rm{Vcontrol}}0)/{\rm{Vcontrol}}0\,}\right]\times 100 \%$$

Randomization was performed using a simple randomization method, ensuring that each mice had an equal chance of being placed in any group.

### Off-target effect assay

Tumor-bearing nude mice were divided into four groups (eight mice per group): vehicle, FGR-KO + vehicle, DCC-2036, and FGR-KO + DCC-2036. Intratumoral injections were administered multiple points twice weekly once tumors exceeded 100 mm^3^. The FGR-KO group received injections of 0.1 ml FGR shRNA lentivirus (5 × 10^8^ TU/ml) and DCC-2036 was administered at 50 mg/kg every other day by gavage.

### Co-culture of CD8^+^ T cells with DKK1-modified LoVo cells

The anti-mouse CD3 antibody (BD Pharmingen, #553057) was applied to a 12-well plate and left to incubate at 4 °C overnight. Purification of CD8^+^ T cells from healthy BALB/c mice spleens was carried out the following day using a 100 µm cell strainer (BD Falcon Cell Strainer, 352360) and LS columns (Miltenyi, 130-042-401) with a magnetic separator (Miltenyi, 130-090-976) and CD8^+^ T cell-specific Microbeads (Miltenyi, 130-117-044). In order to activate the cells, purified CD8 + T cells were grown for 72 hours in RPMI 1640 media that also contained 10% FBS, 1% antibiotics, and 1 µg/mL of mouse CD28 antibody (#553294, BD Pharmingen).

Simultaneously, LoVo cells were either transfected with DKK1-targeting siRNA for knockdown or subjected to DKK1 overexpression for 48 hours. In a 1:5 effector-to-target ratio, activated CD8^+^ T cells were co-cultured with LoVo cells that either had DKK1 overexpressed or were silenced. To be more precise, 1 × 10^5^ LoVo cells were co-cultured with 5 × 10^5^ CD8^+^ T cells in each well for a duration of 48 hours.

Flow cytometry was used to evaluate the CD8^+^ T cells for activity after they had been collected by centrifugation and passed through a 35 µm cell strainer (BD Falcon, 352235).

### Flow cytometry

The co-culture was used to make single-cell suspensions, which were then mixed with 100 µL of PBS and stained using antibodies specific to CD45, CD3, CD4, CD8, and CD69 that were conjugated with fluorescence. The cells were collected by centrifugation at 300 g for 5 min after staining, and aggregates were removed by passing them through a 35 µm cell strainer (BD Falcon, 352235). The samples were then examined using flow cytometry.

The following markers were used to identify and assess activation in T cell subpopulations:

Total T cells (CD3^+^): CD45^+^ CD3^+^

CD4^+^ T cells: CD45^+^ CD3^+^ CD4^+^ CD8^+^

CD8^+^ T cells: CD45^+^ CD3^+^ CD4^−^ CD8^+^

Activated T cells (CD69^+^): CD45^+^ CD3^+^ CD4^−^ CD8^+^CD69^+^

Flow cytometry data were analyzed to evaluate the proportion of CD4^+^ and CD8^+^ T cells, as well as their activation status based on CD69 expression.

### RNA-sequence analysis

RNA-sequence analysis was conducted as previously described [48].

### Immunoprecipitation analysis

Lysates (200 mg) were subjected to immunoprecipitation by being treated with FGR antibodies (Santa Cruz Biotechnology, sc-166079) for one night at 4 °C and with Protein A Sepharose beads (Bimake, #B26201) for three hours. After removing the supernatant, immunocomplexes were examined by western blotting.

### Pull-down assay

Total cell lysates were used for all assays, with protein concentration adjusted to 3–5 mg/ml. For every milliliter of cell lysate, fifty microliters of Pierce^TM^ High Capacity Streptavidin Agarose (#20359, Thermo Fisher Scientific) were added, and then the mixture was incubated on a revolving shaker at 4 °C for two or three hours. Then, for 5 min at 4 °C and 3000 rpm, the mixture was spun in a centrifuge. One milliliter of IP lysis solution containing phosphatase inhibitors (Bimake, #B15001) was used three times to wash the streptavidin agarose beads, followed by one wash with PBS. Mass spectrometry or western blotting analysis followed, after the beads were boiled for 5 min in 2X SDS-PAGE loading buffer (Beyotime, #P0015).

### Luciferase assay

Anhui, China-based General Biosystems (Co., Ltd.) built both the DKK1-WT and DKK1-Mutant promoters. To quantify reporter activity, a luciferase assay kit (#E1910, Promega) was used in accordance with the manufacturer’s instructions. The activity of luciferase was adjusted to match that of the internal control, Renilla luciferase.

### ChIP qPCR

The manufacturer’s instructions were followed while using a ChIP kit (Abcam). Invitrogen provided the SP1 CHIP antibody. The following qPCR primers were used to cover SP1 sites 1 and 2: 5′-CGAGCGACTAAGCAAGGGAG-3′ for the forward primer and 5′-AGACAACAAAGCCGGGATGG-3′ for the reverse primer. Relative enrichment compared to IgG was used to display the results.

### Bioinformatic analysis

Acquired from the UCSC Genome Bioinformatics Site were genome sequences and annotations. Regions ranging from three kilobases upstream to three hundred base pairs downstream of identified transcription start sites were considered promoters. The JASPAR database was used to anticipate SP1 binding locations inside the DKK1 promoter region.

A group of people from The Cancer Genome Atlas (TCGA) were studied. Data visualization was done using the R-package GOplot, while the R-package clusterProfiler was utilized for gene ontology enrichment analysis.

The CIBERSORT tool was employed to estimate the prevalence of various immune cell types, utilizing normalized RNA-seq gene expression data as input files [49].

### Statistical analysis

Unless otherwise specified, all data are expressed as the mean ± structural equation modeling (SEM) and were obtained from diverse samples. The differences observed in sample size did not reach statistical significance. Prism (CA, USA) was employed for the statistical analyses. Based on the characteristics of the data distribution, the Mann-Whitney test, ANOVA, or Student’s t-test was utilized, with subsequent post hoc analyses conducted as required. Significance was established as *P* < 0.05(*), *P* < 0.01(**), *P* < 0.001(***), or *P* < 0.0001(****).

## Supplementary information

Supplementary material

## Data Availability

We admit the availability of data and material. The data discussed in this publication have been deposited in NCBI’s Gene Expression Omnibus and are accessible through the GEO Series accession number GSE214063. The data generated in this study are available in the manuscript and supplementary data files. All data used in this study that were not included in the paper or supplementary files are available upon request from the corresponding author.
