# Supplementary material for: Enhancing immunotherapy efficacy in colorectal cancer: targeting the FGR-AKT-SP1-DKK1 axis with DCC-2036 (Rebastinib)
Source: Cell Death Dis. 2025 Jan 9;16(1):8. doi: 10.1038/s41419-024-07263-8 (PMC11718245; doi:10.1038/s41419-024-07263-8)
Supplement: Supplementary file 1 — Supplementary material [file 41419_2024_7263_MOESM1_ESM.docx]

**Supplementary material**

**Supplementary Figure Legends:**


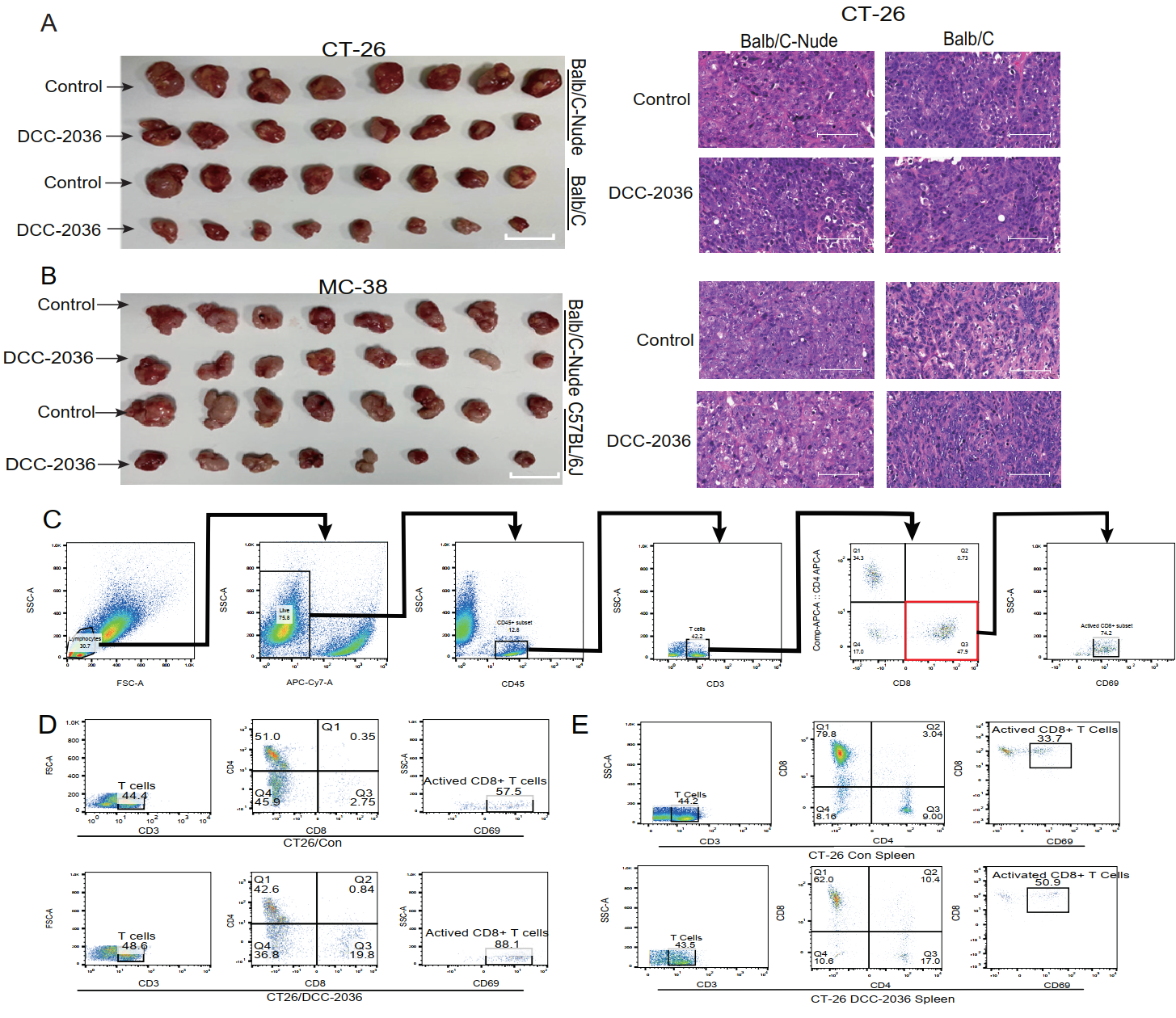


**Figure S1.** **(A)** Ex vivo tumor images in CT-26 transplanted tumor models. Tumor visualization in Balb/C and Balb/C Nude mice treated with DCC-2036 (50 mg/kg) or vehicle control(left). Hematoxylin and Eosin (H&E) staining of tumor tissues. Histological comparison between control and DCC-2036-treated groups showing differences in tumor structure, necrosis, and immune cell infiltration. The DCC-2036-treated group exhibited increased necrosis and a more prominent immune cell presence compared to the control group(right).Treatments were administered via oral gavage every other day. **(B)** Ex vivo tumor images in MC-38 transplanted tumor models. Tumor visualization in C57BL/6J and Balb/C Nude mice treated with DCC-2036 (50 mg/kg) or vehicle control(left). Hematoxylin and Eosin (H&E) staining of tumor tissues. Histological comparison between control and DCC-2036-treated groups showing differences in tumor structure, necrosis, and immune cell infiltration. The DCC-2036-treated group exhibited increased necrosis and a more prominent immune cell presence compared to the control group(right).Treatments were administered via oral gavage every other day. Scale bar = 2.0cm. **(C)** Flow Cytometric Analysis of Live Cells: Logical gating strategy for live cell identification, as depicted in the accompanying graph. **(D)** Lymphocyte Subpopulation Analysis in Tumors. Flow cytometry data showing the distribution of CD8^+^ (%CD3^+^), CD4^+^(% CD3^+^), and CD69^+^(% CD8^+^) T cells in CT-26 xenografts across different treatment groups. **(E)** Lymphocyte Subpopulations in Tumor-Bearing Mice Spleens: Flow cytometry data showing percentages of CD8^+^ T cells (%CD3^+^ cells), CD4^+^ T cells (%CD3^+^ cells), and CD69+ T cells (%CD8^+^ cells).


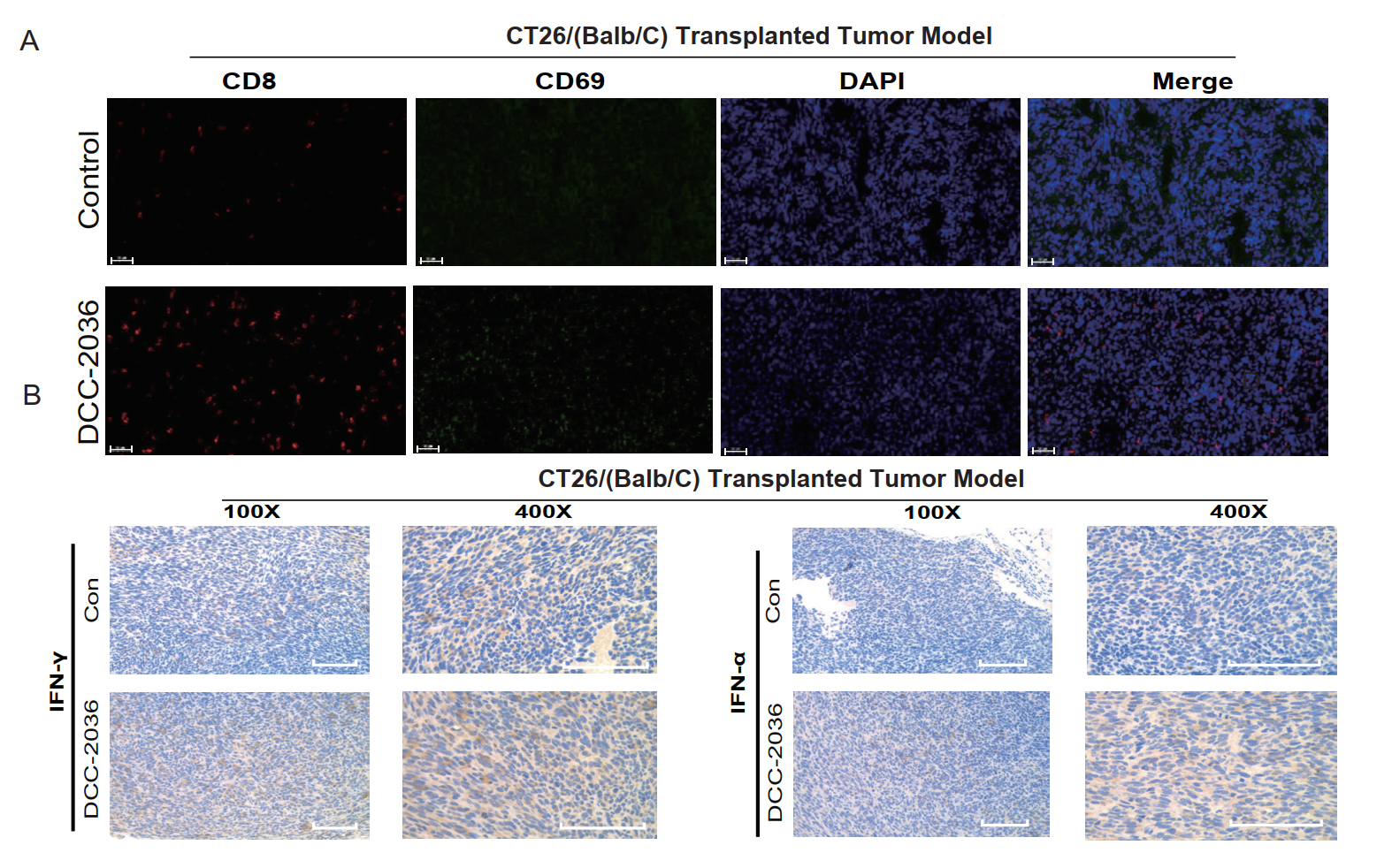


**Figure S2.** (**A)** Immunofluorescence Microscopy for CD8 and CD69, Representative images showing CD8, CD69, and DAPI staining in CT-26 tumors with and without DCC-2036 treatment (scale bars = 50 μm, Left ). (**B)** Immunohistochemistry Staining of IFN-α and IFN-γ: Representative microphotographs (scale bars = 100 μm) depicting staining intensity.


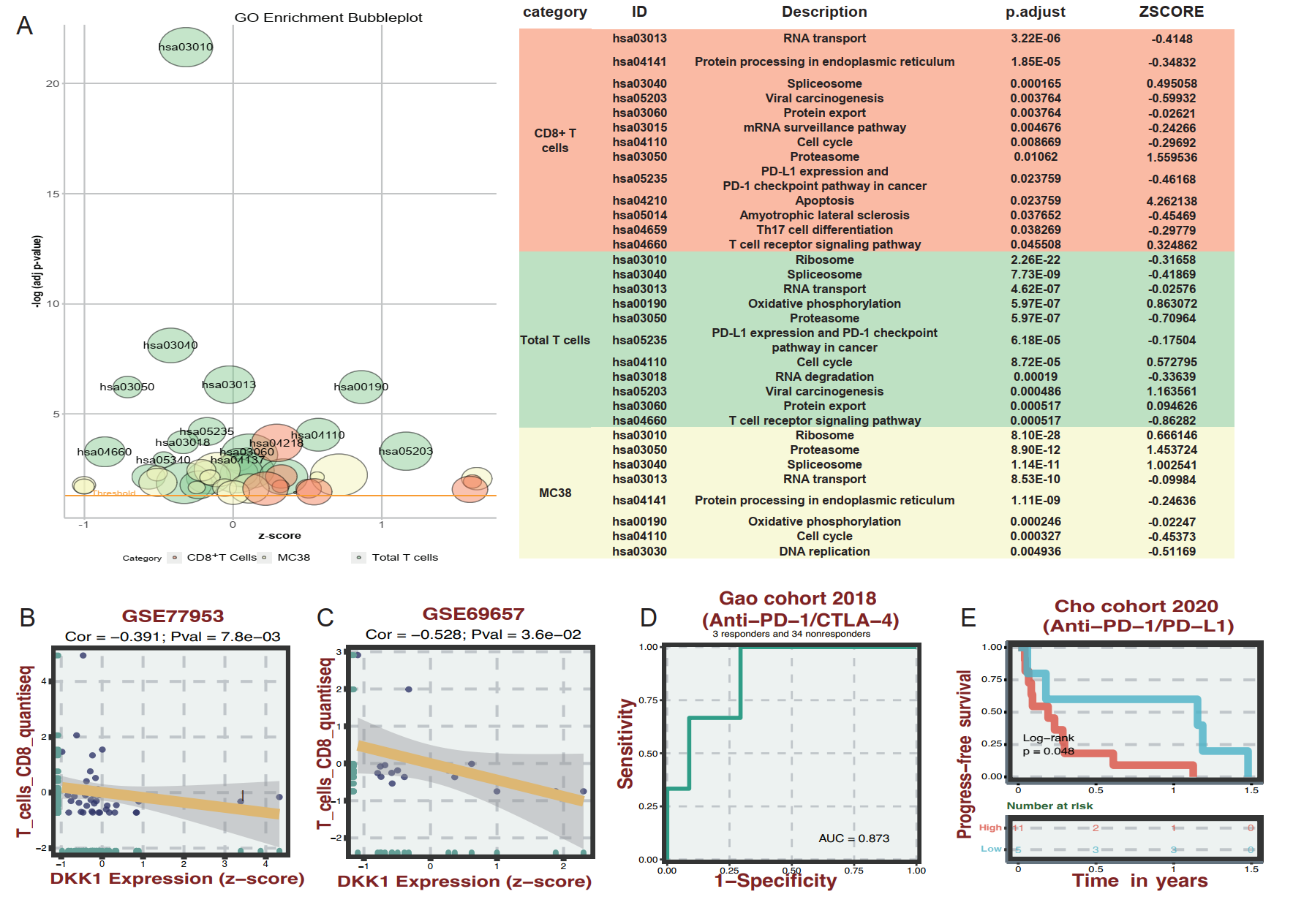


**Figure S3.** **(A)** Gene Ontology (GO) Enrichment Analysis of DKK1-Treated Cells: This bubble plot visualizes the GO enrichment of genes significantly altered in MC-38 cells, T Cells, and CD8^+^ T cells upon DKK1 treatment. The analysis utilizes data sourced from the Gene Expression Omnibus (GEO), series number GSE149206. **(B-C)** Depicts the use of CIBERSORT to predict the correlation between DKK1 expression and CD8^+^ T cells in CRC. The analysis utilizes data sourced from the GEO, series number GSE69657 and GSE77953. **(D)** To demonstrate DKK1's role as a predictive marker for immunotherapy response. *ROC* curves were constructed to assess the specificity and sensitivity of the final prediction models and the area under the *ROC* curve (*AUC*). The analysis utilizes data sourced from the GEO, series number GSE115821. **(E)** Showcases the association of high DKK1 expression with lower PFS after immunotherapy. *PFS* were estimated from initiation of immunotherapy to progression or death (for *PFS*). The analysis utilizes data sourced from the GEO, series number GSE126044.


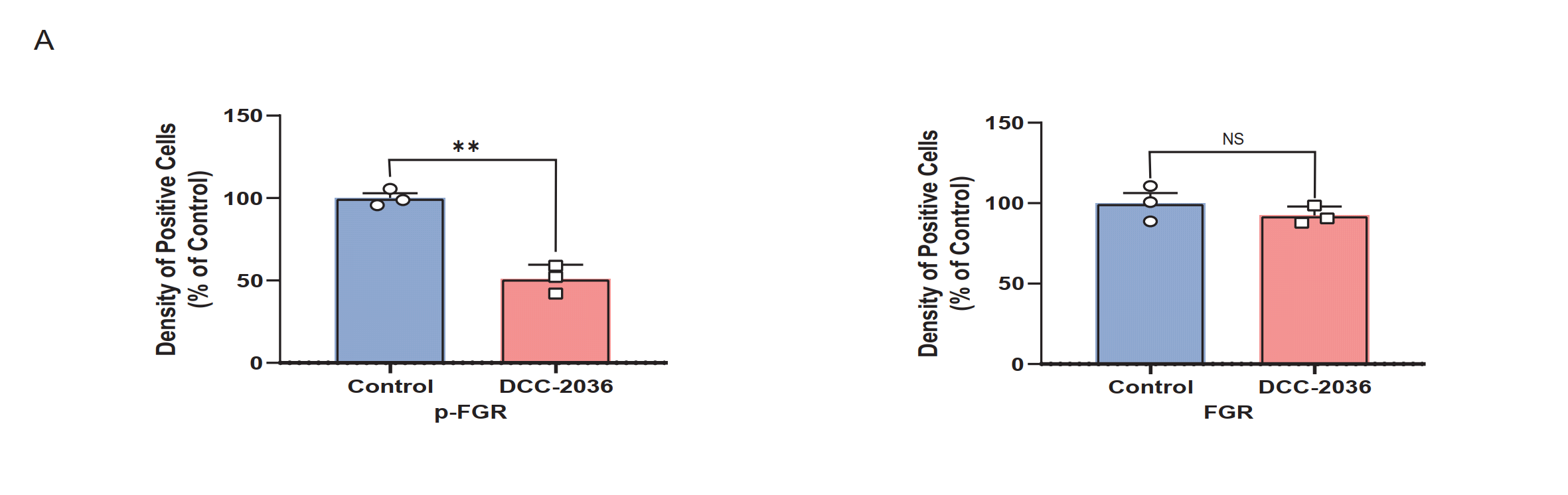


**Figure S4**. **(A)** Immunohistochemical staining was performed to assess the expression levels of p-FGR and total FGR in tumor tissues treated with DCC-2036. The results are presented as the percentage of positive cells compared to control, with densitometric quantification provided. Statistical analysis was conducted using Student's t-test, and the data are shown as mean ± standard deviation. A statistically significant difference was observed for p-FGR (**P < 0.01), while no significant difference was noted for total FGR (ns = not significant).


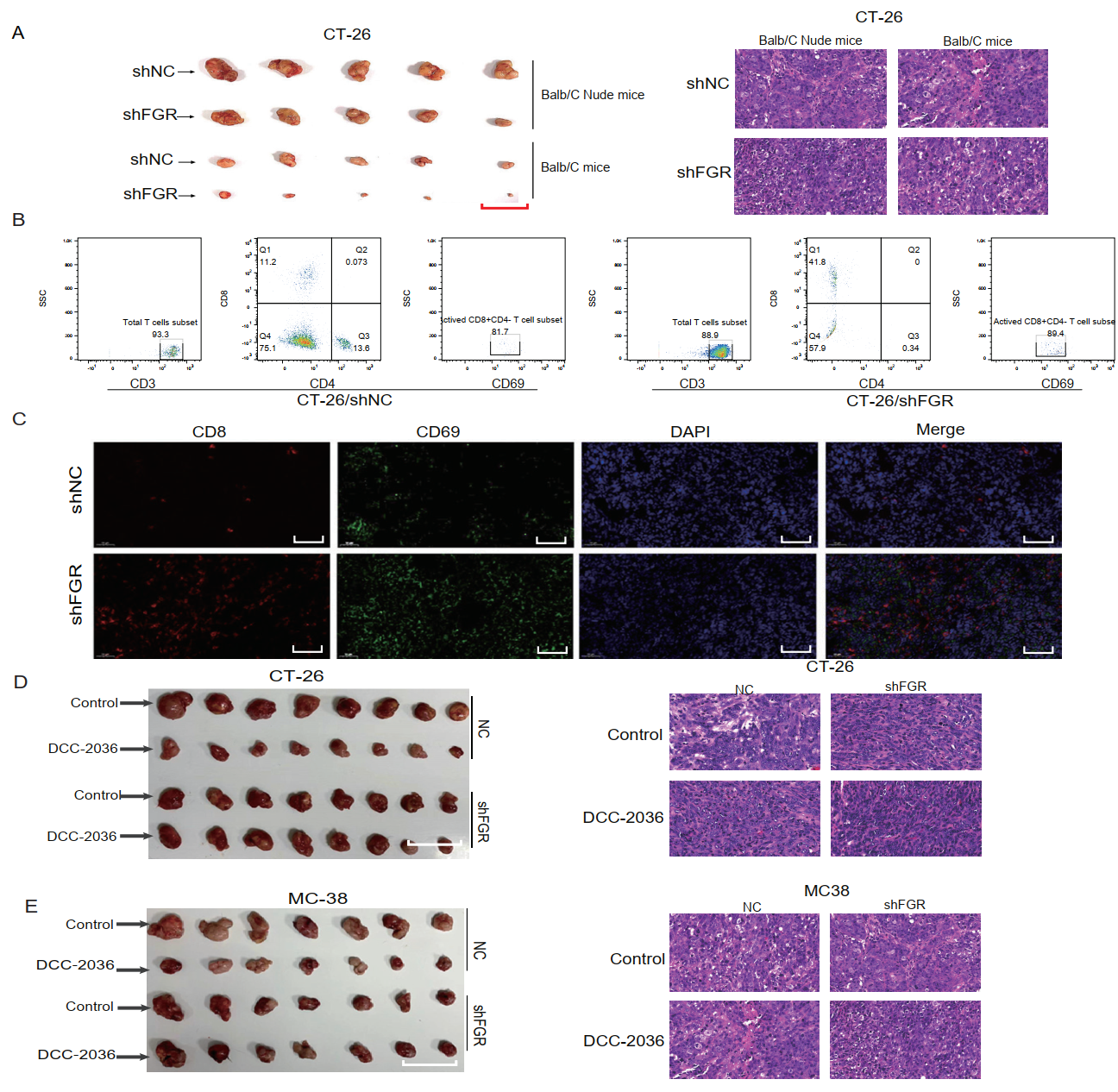


**Figure S5. (A)** Tumor growth of CT-26 homografts generated via intratumor injection of shFGR lentiviruses/Contol. CT-26 cells were subcutaneously administered to 6-week-old Balb/C nude mice and Balb/C mice, with each group consisting of n = 8 mice(left). Hematoxylin and Eosin (H&E) staining of tumor tissues. Histological comparison between control and shFGR lentiviruses injected groups showing differences in tumor structure, necrosis, and immune cell infiltration(right). **(B)** Representative flow cytometry results of lymphocyte subpopulation analysis in implanted tumors of Balb/C mice. Determination of lymphocyte subpopulations within dissected tumors using FCM staining. The proportions of CD8^+^ T cells (% of CD3^+^ T cells) and CD69^+^ T cells (% of CD8^+^ T cells) among the two groups are presented. **(C)** Immunofluorescent Staining in Implanted Tumors of Balb/C mice: The left panel presents representative microphotographs of immunofluorescent staining for CD8, CD69, and DAPI in shNC/shFGR implanted tumors (scale bars = 50 μm). **(D)** CT-26 shFGR/shNC Homograft Mouse Models Treated with DCC-2036: Dissected and photographed tumors(left). Hematoxylin and Eosin (H&E) staining of tumor tissues. Histological comparison between control and DCC-2036-treated groups of shNC/shFGR implanted CT-26 tumors showing differences in tumor structure, necrosis, and immune cell infiltration(right). **(E)** MC-38 shFGR/shNC Homograft Mouse Models Treated with DCC-2036: Similar to (D), Photographs of dissected tumors, CT-26/MC-38 cells, either shFGR or shNC, were subcutaneously injected into mice. These mice were then orally treated with DCC-2036 (50 mg/kg) once every two days. Group size: n = 8. Statistical analysis was performed using Student's t-test for individual comparisons (*p < 0.05, **p < 0.01). Data represent mean ± SD from experiments conducted in triplicate(left). Hematoxylin and Eosin (H&E) staining of tumor tissues. Histological comparison between control and DCC-2036-treated groups of shNC/shFGR implanted MC-38 tumors showing differences in tumor structure, necrosis, and immune cell infiltration(right).


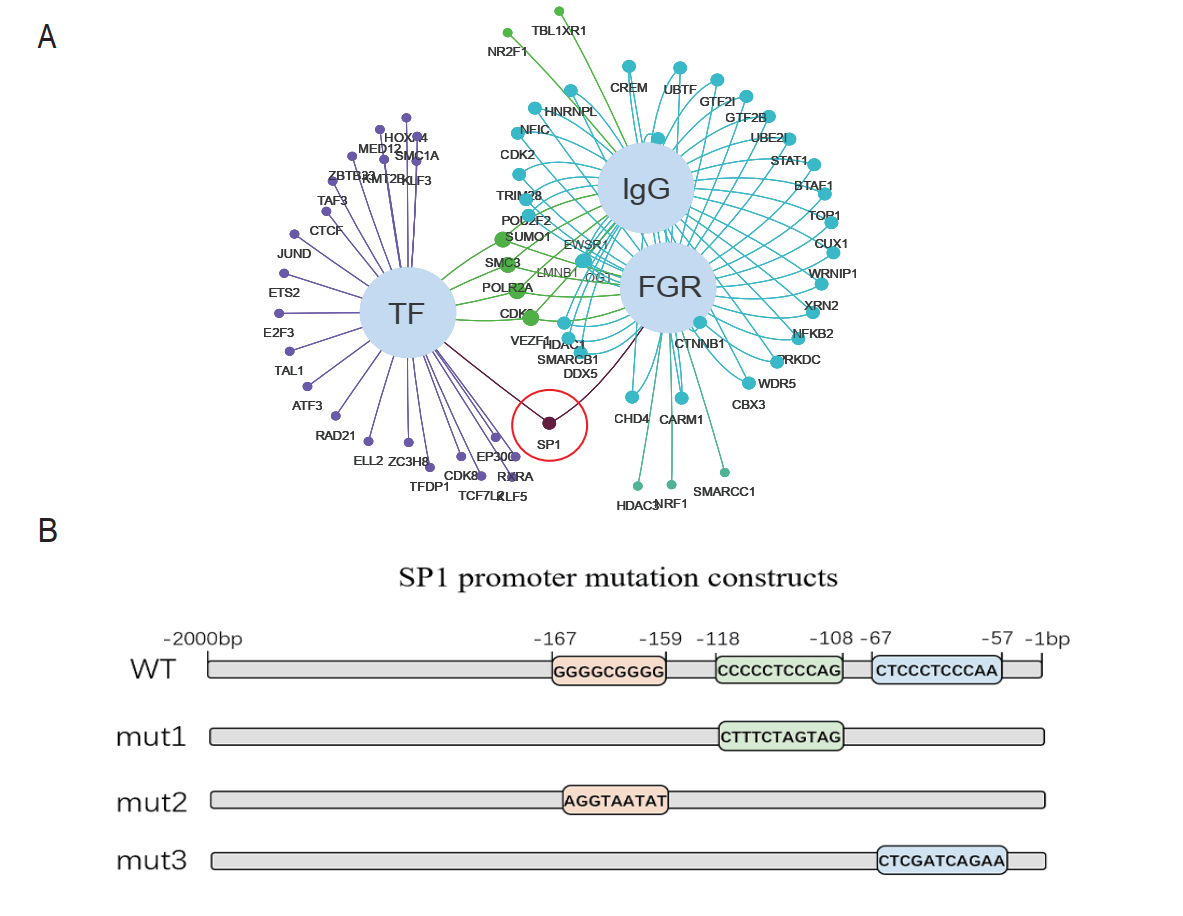


**Figure S6**. **(A)** Network Analysis of FGR Interacting Proteins which can transcriptionally regulate DKK1: Illustration of the protein network interacting with FGR, which is predicted to be transcriptionally active to the DKK1 promoter. Analysis based on Venn diagrams. **(B)** Schematic diagram of DKK1 promoter. The position and sequence of SP1 binding to the DKK1 promoter were predicted using the Jasper database.


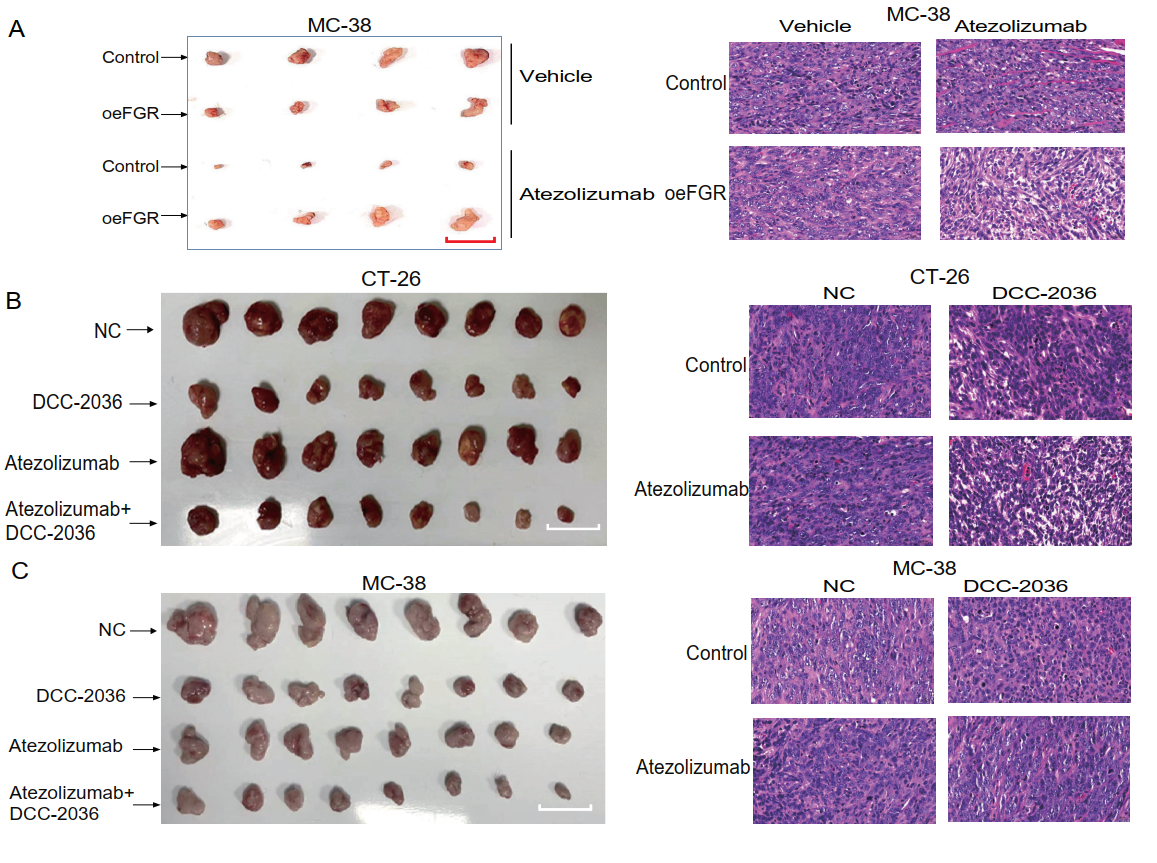


**Figure S7. (A)**Tumor images for MC-38 homografts stably transfected with overexpression FGR lentivirus or control lentivirus, treated with Atezolizumab. MC-38/oeFGR and MC-38/Control cells were subcutaneously injected into congenic mice, which were then orally treated with DCC-2036 as previously described. Group size: n = 4. Scale bar = 2.0cm(left). Hematoxylin and Eosin (H&E) staining of tumor tissues. Histological comparison between control and Atezolizumab -treated groups of Control/oeFGR implanted MC-38 tumors showing differences in tumor structure, necrosis, and immune cell infiltration(right). **(B)** Images of implanted CT-26 subcutaneous tumors in Mice models treated with combinations of DCC-2036 and Atezolizumab or separated in 6–8 week old Balb/C mice. Scale bar = 2.0cm(left). Hematoxylin and Eosin (H&E) staining of tumor tissues. Histological comparison between control, DCC-2036-treated, Atezolizumab -treated or combinations of DCC-2036 and Atezolizumab groups of implanted CT-26 tumors showing differences in tumor structure, necrosis, and immune cell infiltration(right). **(C)** Featuring images of implanted MC-38 subcutaneous tumors in Mice models treated with combinations of DCC-2036 and Atezolizumab or separated in 6–8 week old C57BL/6J mice. Oral administration of DCC-2036 was at 50 mg/kg every other day, and Atezolizumab was injected at 5.0 mg/kg twice a week. Data are from three biological replicates, analyzed using Student's unpaired t-test (*P<0.05, **P<0.01, ***P<0.001) (left). Hematoxylin and Eosin (H&E) staining of tumor tissues. Histological comparison between control, DCC-2036-treated, Atezolizumab -treated or combinations of DCC-2036 and Atezolizumab groups of implanted MC-38 tumors showing differences in tumor structure, necrosis, and immune cell infiltration(right).

**Supplementary Table**

**Table S1. Primers used in the article.**

| Genes | Species | Forward/ Reverse | RNA Sequences(5’-3') |
| --- | --- | --- | --- |
| Fgr | Human | Forward | GCTTCCTTGATAGTGGCACCA |
|  | Human | Reverse | TCAGTTCGAGCCTCATAGTCAT |
|  | Mouse | Forward | GGCTGTGTGTTCTGCAAGAAG |
|  | Mouse | Reverse | GGGGTCAGGGAAATAGCGTT |
| Dkk1 | Human | Forward | CCTTGAACTCGGTTCTCAATTCC |
|  | Human | Reverse | CAATGGTCTGGTACTTATTCCCG |
|  | Mouse | Forward | CAGTGCCACCTTGAACTCAGT |
|  | Mouse | Reverse | CCGCCCTCATAGAGAACTCC |
| Sp1 | Human | Forward | TGGCAGCAGTACCAATGGC |
|  | Human | Reverse | CCAGGTAGTCCTGTCAGAACTT |
|  | Mouse | Forward | AGGGTCCGAGTCAGTCAGG |
|  | Mouse | Reverse | CTCGCTGCCATTGGTACTGTT |
| Gapdh | Human | Forward | TGACTTCAACAGCGACACCCA |
|  | Human | Reverse | CACCCTGTTGCTGTAGCCAAA |
|  | Mouse | Forward | AGGTCGGTGTGAACGGATTTG |
|  | Mouse | Reverse | GGGGTCGTTGATGGCAACA |

**Supplementary Methods**

Fgr shRNA Lentivirus

| **NO.** | **Target Seq** |
| --- | --- |
| Fgr-RNAi(100090-1) | ctATGGTAGCTTGCTGGATTT |
| Fgr-RNAi(100091-1) | cgGCACTACATGGAAGTGAAT |
| Fgr-RNAi(100092-1) | ctTCGGAAAGATCAGTAGAAA |
| Description | Mus musculus FGR proto-oncogene, Src family tyrosine kinase (Fgr), mRNA |

Fgr small interfering RNA (siRNA)

| **NO.** | **Target Seq** |
| --- | --- |
| Fgr-Homo-320 | ctATGGTAGCTTGCTGGATTT |
| Fgr-Homo-437 | cgGCACTACATGGAAGTGAAT |
| Fgr-Homo-758 | ctTCGGAAAGATCAGTAGAAA |
| Description | Homo sapiens FGR proto-oncogene, Src family tyrosine kinase (Fgr), mRNA |

**Enzyme-linked immunosorbent assay (ELISA)**

ELISA was carried out in accordance with instruction of the ELISA Kit. In brief, isolated supernatant samples (10^3^ times dilution), bio-antibody, and streptavidin-HRP were added into 96-well plates pre-coated with antibodies and incubated at 37 ℃, respectively. After 60 min, the liquid was discarded and washed thoroughly. Next, chromogen solution was poured into each well and preservation for 15 min at 37 ℃ to evade the light. Finally, stop solution was infused into each well, and absorbance was measured at 450 nm. The Control group was conducted without supernatant. Each sample was measured in three repetitive experiments.

**Mass spectrometry**

Mass spectrometry analysis was carried out by LC−MS−MS using an LTQ-Velos-Orbitrap (Thermo Electron) connected to a nanoLC Ultimate 3000 Rapid Separation Liquid chromatography system (Dionex). A volume of 5 μl corresponding to 20% of the whole sample was injected into the system. After preconcentration and washing of the sample on a Dionex Acclaim PepMap 100 column (C18, 2 cm × 100 μm i.d. 100 Å pore size, 5 μm particle size), peptides were separated on a Dionex Acclaim PepMap RSLC column (C18, 15 cm × 75 μm i.d., 100 Å, 2 µm particle size) at a flow rate of 300 ml/min−1 with a two steps linear gradient (4–20% acetonitrile/H_2_O; 0.1% formic acid for 90 min and 20–45% acetonitrile/H_2_O; 0.1% formic acid for 30 min). The separation of the peptides was monitored by a UV detector (absorption at 214 nm). For peptide ionisation in the nanospray source, the spray voltage was set at 1.4 kV and the capillary temperature at 275 °C. All samples were measured in a data-dependent acquisition mode. The peptide masses were measured in a full survey scan (scan range 300–1700 m/z, with 30 K FWHM resolution at m/z = 400, target AGC value of 1 × 10^6,^ and maximum injection time of 500 ms). In parallel to the full high-resolution scan in the Orbitrap, the data-dependent CID scans of the 10 most intense precursor ions were

fragmented and measured in the linear ion trap (normalized collision energy of 35%, activation time of 10 ms, target AGC value of 104, maximum injection time 100 ms, isolation window 2 Da). Parent masses obtained in the orbitrap analyzer were automatically calibrated on the 445.120025 ions used as lock mass. The fragment ion masses were measured in the linear ion trap to have maximum sensitivity and the maximum amount of MS/MS data. Dynamic exclusion was implemented with a repeat count of one and an exclusion duration of 30 s. Each sample was analyzed in triplicate on the mass spectrometer. Each run was preceded by a blank MS run in order to monitor the system background.

**Western blotting**

For Western blot analyses, Whole lysates were prepared with radioimmunoprecipitation assay (RIPA) buffer. The cytosolic and nuclear fractions were prepared with a Nuclear Extraction Kit (Merck Millipore) according to the manufacturer's protocol. The prepared protein samples (60-100mg) were separated by SDS-PAGE, electro-transferred onto polyvinylidene difluoride membranes (Millipore), and probed overnight with antibodies, then proteins were visualized by horseradish peroxidase-conjugated IgG antibodies and ECL SuperSignal (Millipore, #WBKLS0050) exposed to X-ray film. All samples were normalized to a control sample on each gel, and phosphorylation levels were additionally normalized to total protein after membranes were stripped, as previously reported, and reprobed with the corresponding antibody for total protein.

**Real-time RT-PCR**

Total RNA was extracted from cells using TRIzol reagent (#15596026, Invitrogen; Thermo Fisher Scientific, USA). Isolated RNA was reverse transcribed using the RevertAid First Strand cDNA Synthesis Kit (Thermo Fisher Scientific) according to the manufacturer's protocol. The following primers were used(Table S1)

Quantitative measurement of target gene mRNA levels was performed using the ABI Prism 7500 Sequence Detection System (Applied Biosystems; Thermo Fisher Scientific). The data were analyzed using the 2-ΔΔCq method.

**Preparation of** **single-cell suspension**

After treatment, tumor tissues, and spleen were made into single-cell suspensions. Prepare enzyme mix by adding 2.7 mL of DMEM, 200 µL of Collagenase I (Solarbio, C8140), and 100 µL of Collagenase II (Solarbio, C8150) into a gentleMACS C Tube. Biopsies were collected in RPMI 1640 media supplemented with penicillin/streptomycin and immediately transferred for dissociation within minimum ischemic time. On arrival, each sample was placed in a sterile Petri dish on ice. Fat, fibrous, and necrotic areas were removed, and the tissue was subsequently minced into smaller pieces of less than 3 mm. Sample pieces were transferred to a gentleMACS C Tube (Miltenyi #130-093-237) containing 5 ml digestion enzyme mix previously prepared according to the kit user guide (Tumor dissociation Kit from Miltenyi Biotech # 130-093-235). The C tube was then placed on a gentleMACS™ Dissociator for mechanical dissociation using the program m_impTumor_02. Incubate sample for 40 minutes at 37°C with continuous rotation using the MACSmix Tube Rotator. Attach C Tube upside down onto the sleeve of the gentleMACS Dissociator. Run the gentleMACS Program m_impTumor_03. Upon run completion, the sample was incubated for 30 min at 37 °C under continuous low shaking. Subsequently, the sample was filtered using a 70 μm nylon mesh (Miltenyi # 130-095-823). 10 μl of this cell suspension was counted by Trypan Blue to determine the concentration of live cells. The sample was then centrifuged at 300×g and 4 °C for 5 min, and the supernatant was discarded. Throughout the dissociation procedure, cells were maintained on ice whenever possible, and the entire procedure was completed in less than 1 h.

**Hematoxylin and eosin staining / IHC / IF staining**

One hundred paraffin-embedded colorectal cancer samples were collected from the Department of Pathology, Hunan Cancer Hospital (Changsha, China), and made into the high-density colorectal cancer tissue microarray slides by Servicebio (Wuhan, China).

In detail, Serial 4 μm sections were used for hematoxylin and eosin, IHC, and IF staining. Antibodies were diluted in Antibody Diluent Reagent Solution (90% PBST+10% goat serum, Absin, abs933) and reactions were carried out overnight at 4 ℃. After washing with PBS three times (5 min each time), the sections were incubated with horse anti-mouse antibody (Absin, abs997) at room temperature for 30 min. After washing three times with PBS, the slides were stained with 2-(4-aminophenyl)-6-indolecarbamidine dihydrochloride (DAPI) for 2 min to show the locations of nuclei. Images were acquired using an Olympus BX40 microscope (Olympus Corporation, Tokyo, Japan). The Image-pro Plus 5.0 (Media Cybernetics, Inc. Rockville, MD, USA) was applied to calculate the expression.

**Antibody array**

For RTK phospho-protein array analysis, cell culture lysates were prepared and analyzed using a Pathscan RTK Signaling Antibody Array kit (Cell Signaling Technologies). Briefly, 100 µg lysate was loaded on an array membrane, incubated overnight at 4 °C, and then washed with PBS/0.05% Tween, serially incubated for 1 h with the detection biotinylated antibody (150 μl/well; 1 × dilution) and DyLight-680-conjugated streptavidin (150 μl/well, 1 × dilution). Arrays were imaged using an Odyssey Infrared Imaging System and spot intensities were quantified using Image Studio software.


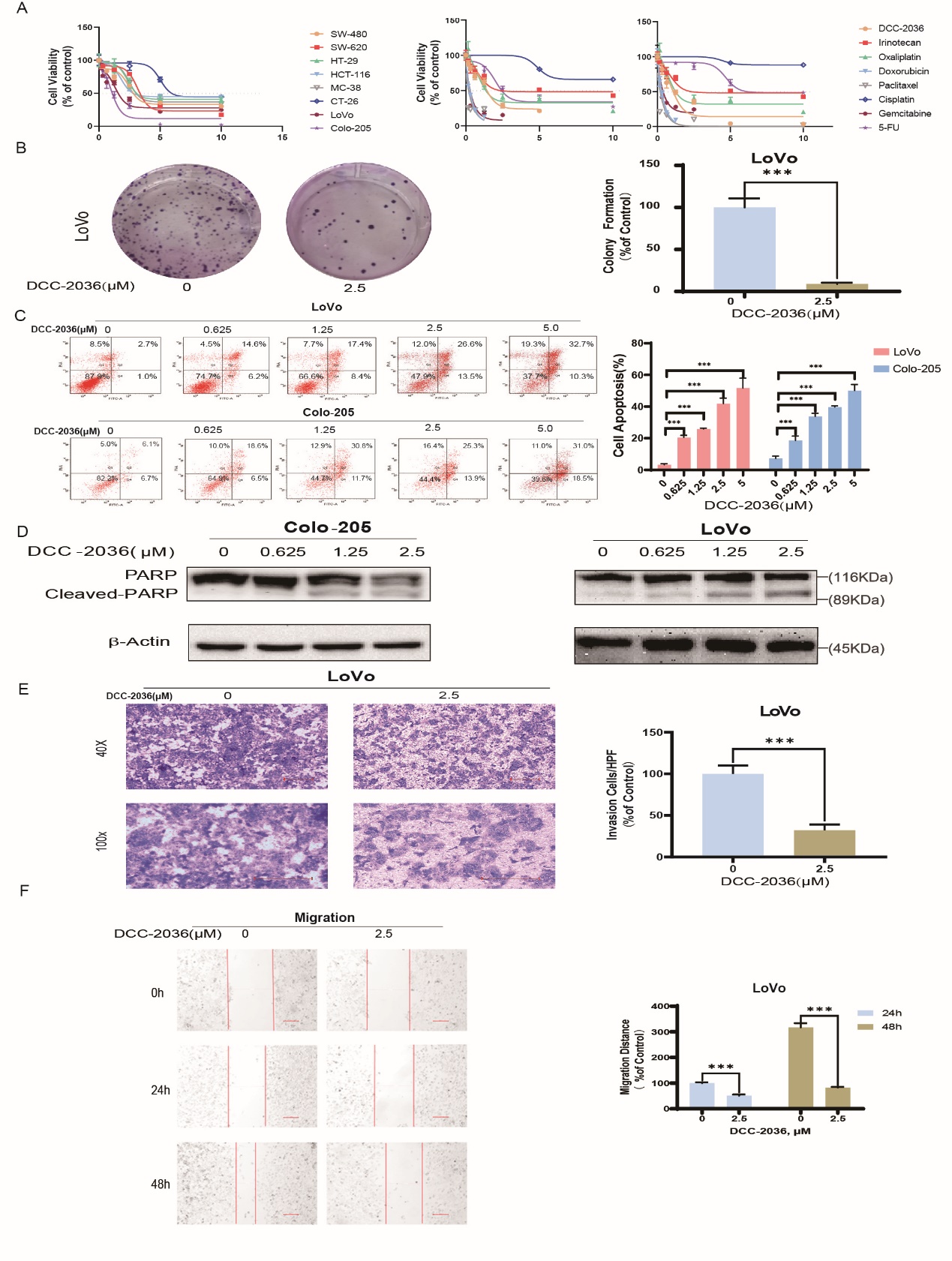


**Figure E1. Anticancer Effects of DCC-2036 in Colorectal Cancer Cell Lines. (A)** MTS assays were performed after 72 h treatment by DCC-2036 in SW-480, SW-620, HT-29, HCT-116, MC-38, CT-26, LoVo, Colo-205 cells (Left). Cytotoxicity of DCC-2036, Irinotecan, Oxaliplatin, Doxorubicin, 5-FU, Paclitaxel, Cisplatin, Gemcitabine in LoVo and Colo-205 cells were also assessed by MTS assay (Right). IC50 values were measured by GraphPad Prism software 9.1. (**B)** Images of colony formation assay in 6-well plates after exposure to 0 μM or 2.5μM DCC-2036. 2000 viable cells after DCC-2036 treatment were plated in 6-well plates in drug-free DMEM. After 14 days, the number of colonies was counted. Left, representative graphs of three independent experiments; right, statistical charts. Columns, mean; bars, SD. Student's t-test. *** P <0.001. (**C)** Induced Apoptosis was determined by flow cytometry (FCM) with AnnexinV-FITC/PI dual staining. Representative Flow cytometry data (left) and statistical graph (right) were shown. Representative of three independent experiments; right, statistical charts with the vertical axis representing the sum of all dead cells, except the left lower quadrant; *** P < 0.001, Student t-test. **(D)** Levels of Cleaved-PARP in LoVo and Colo-205 cells after DCC-2036 treatment were examined by Western blotting, the β-ACTIN was used as an internal reference. (**E)** Effect of DCC-2036 treatment on cell invasion by transwell assay. 5×10^4^ viable cells after DCC-2036 treatment were plated in 24-well transwell chambers embedded with Matrigel, and invasive cells on polycarbonate transwell membrane were photographed and counted under a microscope (40x, 100×) (top). The percentage of invasive cells from three independent experiments was shown (bottom). (**F)** Wound healing scratch assays were performed to indicate the inhibition of DCC-2036 on cell migration. 2.5×10^5^ viable cells after DCC-2036 treatment were plated in 6-well plates. The scratched wound was photographed at 0, 24, and 48 h (left). The percentage of migrated cells from three independent experiments was shown(bottom). Columns, mean; error bars, SD. Student’s t-test, *** P <0.001(right).


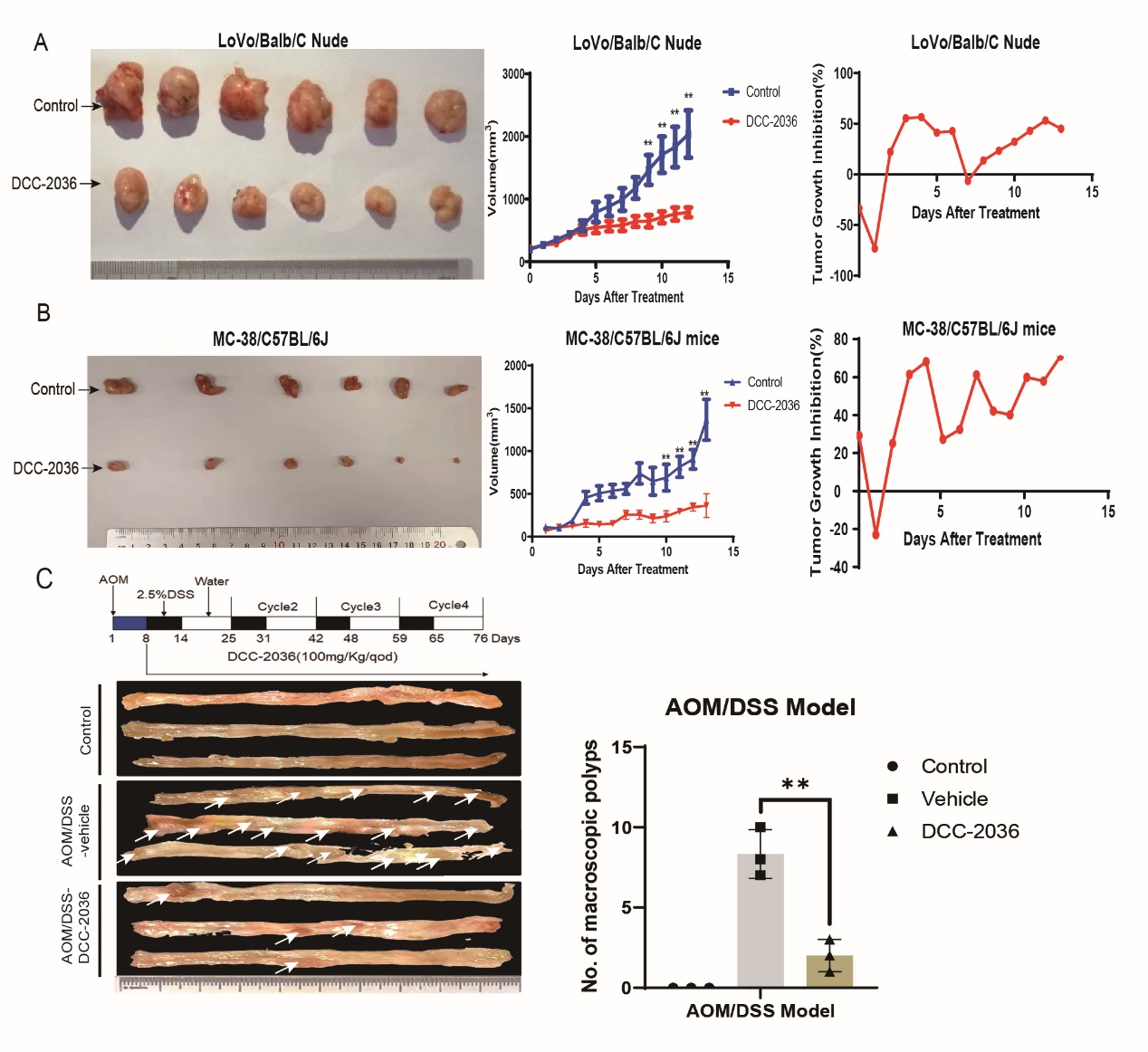


**Figure E2. Effects of DCC-2036 on Tumor Growth and Inhibition in Xenograft and Colitis-Associated Colon Cancer Models. (A)** Ex vivo tumor images, growth curves, and tumor inhibitory curves of LoVo (Balb/C Nude mice) subcutaneous tumor xenograft models treated with/without DCC-2036. Inoculating 6-week-old mice with LoVo (Balb/C Nude mice) cells subcutaneously and administered with DCC-2036 (100 mg/kg) or vehicle control (0.5% carboxymethyl cellulose (CMC)+1% Tween 80) through oral gavage every other day (QOD). (**B)** Ex vivo tumor images, growth curves, and tumor inhibitory curves of MC-38 (C57BL/6J mice) subcutaneous tumor xenograft models treated with/without DCC-2036. Inoculating 6-week-old mice with LoVo (Balb/C Nude mice) cells subcutaneously and administered with DCC-2036 (100 mg/kg) or vehicle control (0.5% carboxymethyl cellulose (CMC)+1% Tween 80) through oral gavage every other day (QOD). **(C)** Schematic representation of experiment design for AOM/DSS-induced colitis-associated colon cancer in C57BL/6J mouse (top). Images of colonic tumors (bottom) with or without DCC-2036 treatment. The statistic chart of the colon cancer counts, student’ t-test, **, P <0.01.
